# Navigating the landscape of direct cellular reprogramming with DiReG

**DOI:** 10.1038/s41540-026-00652-z

**Published:** 2026-02-06

**Authors:** Michael Lauber, Markus List

**Affiliations:** 1 Data Science in Systems Biology, TUM School of Life Sciences, Technical University of Munich, Freising, Germany; 2 Munich Data Science Institute (MDSI), Technical University of Munich, Garching, Germany

**Keywords:** Computational biology and bioinformatics, Mathematics and computing

## Abstract

Direct cellular reprogramming, converting one differentiated cell type directly into another, holds immense promise for regenerative medicine, developmental biology, and disease modeling. Identifying optimal transcription factor (TF) combinations to control this process remains complex and labor-intensive. Over the last decade, various computational tools emerged to infer TF sets for reprogramming. However, current methodologies possess critical limitations, and the absence of robust benchmarking standards makes it impossible to precisely validate and compare their performance. To address these challenges, we present a comprehensive analysis of existing computational methods for direct reprogramming and introduce a web application designed to support researchers in identifying and validating optimal TF sets. Our platform integrates predictions from established tools, incorporates a state-of-the-art Retrieval-Augmented Generation (RAG) system for efficient literature querying, and offers tools to further validate predictions. By providing a unified and interactive resource, our web application enhances the accessibility and efficiency of TF discovery for direct reprogramming. Furthermore, we discuss critical limitations shared by current methodologies and highlight the need for computational tools that can account for the complex regulatory dynamics of direct reprogramming. This work not only advances the toolkit available to researchers but also lays the groundwork for future innovations aimed at realizing the full potential of direct reprogramming.

## Introduction

Almost four decades ago, Davies et al. discovered that the ectopic expression of the transcription factor MyoD1 is sufficient to convert fibroblasts into stable myoblasts^1^. This was the first demonstration that a single transcription factor could induce direct lineage conversion and provided the first molecular evidence for direct reprogramming or transdifferentiation.

Direct reprogramming refers to the direct conversion of one fully differentiated cell type into another through the action of specific transcription factors (TFs) or external cues, bypassing a pluripotent or progenitor state^2^. After MyoD’s potential for transdifferentiation was discovered, more protocols for generating other specialized cell types, such as neurons^3^ or insulin-producing beta cells^4^ emerged and opened new avenues in regenerative medicine, developmental biology, and disease modeling.

In 2006, Shinya Yamanaka demonstrated that somatic cells could be reprogrammed into a pluripotent state by introducing four TFs: Oct4, Sox2, Klf4, and c-Myc^5^. These reprogrammed cells, termed induced pluripotent stem cells (iPSCs), enable the generation of specialized cell types through a pluripotent intermediate stage. iPSC-based reprogramming allows for creating cells with indefinite self-renewal capacity and opens the potential to differentiate into any cell type under appropriate conditions. Unlike transdifferentiation, iPSC-based reprogramming resets the cell’s epigenetic age, making it advantageous for applications where studying age-related phenotypes is not a primary focus^6^. However, the multi-step nature of this process, involving reprogramming to pluripotency followed by differentiation into the desired cell type, may limit its feasibility for direct in vivo applications due to complexities in cell fate control and potential tumorigenicity.

While these biological breakthroughs laid the foundation for reprogramming research, discovering reprogramming protocols has traditionally relied on expert-driven hypotheses and labor-intensive wet lab experiments, including combinatorial transcription factor screening and gene expression profiling. Despite significant advances, generating fully functional and mature cell types remains challenging due to incomplete epigenetic resetting, unstable cell states, and limited understanding of developmental signaling pathways^7,8^. Critical targets such as long-term repopulating hematopoietic stem cells and fully glucose-responsive beta cells remain elusive due to these unresolved barriers.

To address these challenges, computational tools leveraging systems biology, machine learning, and gene regulatory network modeling have emerged during the last decade. By predicting optimal transcription factor combinations, these tools aim to reduce the reliance on large trial-and-error experimentation and accelerate the development of more efficient protocols. While still evolving, such methods hold promise for overcoming current limitations and moving directed reprogramming closer to clinical applications.

In this publication, we begin by analyzing the current state-of-the-art computational tools for direct reprogramming, detailing their algorithmic foundations, capabilities, and specific limitations. We then demonstrate to the reader that no existing method can currently be considered the gold standard due to the lack of fair and independent benchmarking. Furthermore, we address limitations common to all computational approaches that have yet to be tackled but are essential for developing successful future methods.

Subsequently, we introduce an interactive Directed Reprogramming Guide (DiReG, available at https://daisybio.ls.tum.de/direg/app/direg) designed to help researchers identify optimal transcription factor sets.

DiReG is not proposed as a superior de novo prediction algorithm intended to replace existing methods. Rather, it functions as an integrative platform designed to bridge the gap between computational prediction and experimental application. It serves four primary goals: (1) acting as a common resource to compare precomputed predictions from various tools, (2) providing a high-accuracy RAG (Retrieval-Augmented Generation) for answering questions derived from reprogramming publications, (3) providing an easy and quick way of inference based on epigenome data and (4) offering a procedure to validate the potential of reprogramming cocktails using a diverse set of metrics. This validation process enables scientists to test hypotheses driven by expert knowledge easily, granting access to various tools and databases and bridging the gap until next-generation computational tools become available.

Finally, we provide an outlook on the key advancements required for the next generation of bioinformatics approaches to enhance protocol discovery.

## Results

### Computational tools for direct reprogramming

**Fig. 1:**
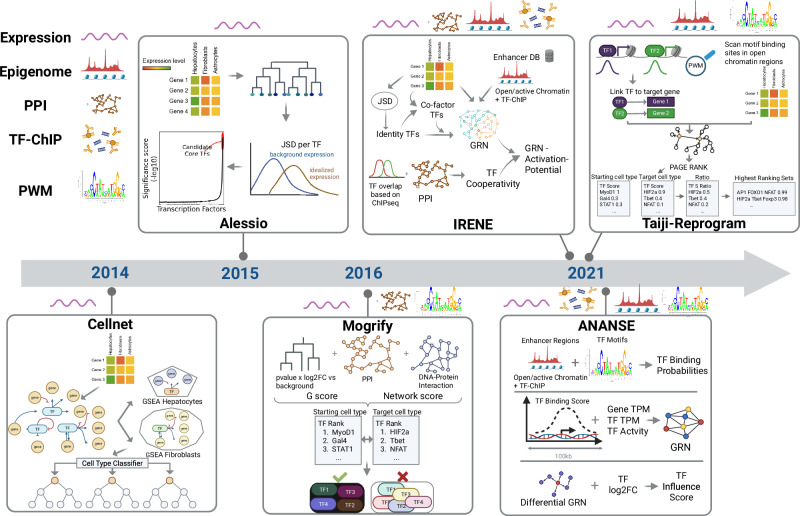
A chronological timeline of computational tools developed to predict TFs for direct cell reprogramming. Each tool is represented by a box containing a schematic illustration of its core methodology and the primary data types it incorporates (gene regulatory networks, epigenomic profiles, protein–protein interactions, or transcriptomic data). The timeline demonstrates the field’s progression from early network-based approaches to advanced integrative methods that combine multiple data modalities for improved prediction of transcription factor combinations in cellular conversion. Figure created with BioRender.com.

In this section, we introduce six methods specifically designed to discover reprogramming TFs (Table 1). For each method, we explain its inner workings in detail, enabling readers to understand the method’s premise, capabilities, and inherent limitations. To facilitate comprehension of the methods’ progression, they are presented in chronological order of publication (Fig. 1). Finally, we provide a brief section introducing alternative methods that, while not explicitly designed for identifying reprogramming TFs, can be repurposed for this aim.

**Table 1:** Comparison of computational methods for identifying transcription factors in direct reprogramming, highlighting data requirements, code availability, algorithmic strategy, strengths, and limitations

Method	RNAseq	ChIP	Epigenome	Code	Strategy	Strengths	Limitations
CellNet	✔	✘	✘	GitHub	Mutual information-based GRN construction; random forest classifier	Evaluates similarity between reprogrammed and target cells; identifies candidate TFs	Expression data only; ignores TF interactions; no optimized TF combinations provided
D’Alessio et al.	✔	✘	✘	N/A	Entropy-based Jensen-Shannon Divergence (JSD) scoring	Adaptable to new datasets; outputs clear list of core TFs	No code available; assumes that expression equals activity
Mogrify	✔	✘	✘	N/A	Diff. expression combined with regulatory influence (PPI & DNA)	Multi-modal data integration; filters out redundant TFs	Generic, non-cell-specific PPI/DNA data; uses outdated datasets (e.g., FANTOM5)
IRENE	✔	✔	✔	GitHub	JSD scoring with epigenomics and boolean logic	Incorporates epigenomic data; models TF cooperativity	High data dependency; static logic rules; user must pre-define the number of factors to predict
Taiji- Reprogram	✔	✔	✔	GitHub	Personalized PageRank algorithm	Incorporates epigenomic data; scalable graph-based algorithms	Bias toward highly connected TFs; may miss master regulators with lower connectivity
ANANSE	✔	✔	✔	GitHub	Enhancer-centric approach integrating TF binding, activity, and expression data	Captures tissue specificity via enhancers; models promoter-enhancer interactions.	Ignores TF cooperativity

CellNet, developed by Cahan et al. in 2014, is a network biology-based approach that evaluates how closely reprogrammed cells resemble their intended target cell types and identifies candidate TFs to improve reprogramming^9,10^. The method constructs a general gene regulatory network (GRN) from gene expression data from various cell and tissue types using mutual information-based algorithm^11^ to identify relationships between genes. A community-detection algorithm (Infomap^12^) partitions the GRN into smaller subnetworks based on gene connectivity patterns.

Each subnetwork undergoes Gene Set Enrichment Analysis (GSEA), linking it to Gene Ontology Biological Processes associated with specific cell or tissue types to identify subnetworks characteristic of distinct cell types. A random forest classifier trained on the cell type-specific subnetworks provides feature importance scores for individual genes which form the basis of a GRN Status (GS) score. For a query sample, the GS score represents the cumulative sum of feature importance values, weighted by gene expression relative to the reference distribution in the target cell type. CellNet also estimates a Network Influence Score (NIS) for each TF reflecting both the TF’s expression and its regulatory impact on target genes within the GRN. Comparing NIS values between the query and target cell types highlights TFs that could improve the reprogramming process.

As one of the first algorithms designed to assist scientists in cellular reprogramming, CellNet has notable limitations. The algorithm relies solely on expression data, and its dependence on mutual information often results in TFs being associated with highly correlated genes that may, in reality, be regulated by other TFs. Additionally, although CellNet proposes an NIS for TFs, it neither accounts for interactions between these TFs, nor provides an optimized set of TFs for reprogramming purposes.

Shortly after Cellnet appeared, D’Alessio et al. developed a computational approach to identify core TFs for direct cellular reprogramming^13^. Their premise is that core TFs, a small set of TF capable of establishing cell-type-specific expression patterns, tend to be expressed in a cell-type-centric manner and at high levels.

The authors gathered approximately 500 expression datasets representing 106 human cell and tissue types, drawn primarily from the Human Body Index collection. To reduce redundancy and balance this background dataset, they performed hierarchical clustering based on pairwise Pearson correlation coefficients of gene expression profiles, and selecting the medoid as each cluster’s representative. To assess cell-type specificity, the authors applied an entropy-based Jensen-Shannon Divergence (JSD) measure^14^. JSD quantifies how far a TF’s observed expression pattern deviates from an idealized pattern of exclusive expression in the query cell type. This scoring method integrated both the level of expression and its specificity to rank candidate core TFs for each cell type.

Compared to CellNet, D’Alessio et al.’s algorithm provided scientists with a precise list of core TFs. The approach remains limited by its exclusive reliance on gene expression data and the simplified assumption TF’s expression directly correlates with its regulatory activity.

Rackham et al. introduced Mogrify in 2016, an approach that combines gene expression data with regulatory network information to predict TFs for direct programming^15^. Mogrify calculates a differential expression score G for each TF per cell-type by combining the log-transformed fold change with the adjusted *p*-value, computed relative to a background of distantly related cells. The tool then models each TF’s regulatory influence using two distinct networks: one based on protein–DNA interactions and another on protein–protein interactions (PPI) data.

For each TF in a given cell type, Mogrify calculates a weighted sum of the G scores of its target genes. Directness is incorporated by assigning higher weights to targets reached through shorter regulatory paths, emphasizing more immediate regulatory effects. Specificity is addressed by weighting TFs with fewer downstream targets more heavily, reducing the influence of broad regulators. Based on the local neighborhood scores and the G score alone, ranked lists are calculated per cell type. Each list is limited to the top 100 results, and an average rank is calculated for each TF. To generate predictions ranked lists of the starting and target cells are compared: TFs ranking low in the starting cell but high in the target cell are flagged as potential candidates. TFs that sharing common targets with a lower-ranked TF are removed.

Mogrify represented a clear advance over earlier methods by integrating multiple data modalities. The protein–protein and protein–DNA interaction data used, however, were not cell type-specific, likely introducing many false-negative and false-positive interactions.

In 2021, Jung et al. proposed the Integrative Gene Regulatory Network Model (IRENE)^16^ extending D’Alessio et al.’s approach by incorporating epigenomic data alongside transcriptomic data and drawing PPI information to predict TF sets for cellular conversions.

IRENE begins by calculating a set of 10 TFs with the highest JSD, termed identity TFs, using a modified version of D’Alessio et al.’s method. The modification simplifies background data generation by excluding data above a Pearson correlation coefficient cutoff. Cell-type-specific core GRNs are then constructed by classifying TFs as active or inactive based on their expression levels. For each active TF, promoter and enhancer regions (identified from GeneHancer) are similarly classified as active or inactive based on histone modifications. Active regulatory regions showing cell-type-specific DNase peaks are scanned for TF binding sites using ChIP-seq data from ChIP-Atlas.

From these data, a cell-type-specific core GRN scaffold is constructed, including only identity TFs and co-factor TFs. Co-factor TFs are defined as active, cell-type-enriched TFs regulated by at least one identity TF.

IRENE determines cooperative and competitive TF binding by analyzing overlapping ChIP-seq peaks with reported PPIs, applying Boolean logic inference. The model predicts efficient TF combinations by evaluating their ability to activate the entire core GRN and assessing the fraction of active regulatory elements after TF overexpression.

IRENE stands out as the first algorithm to consider TF cooperativity and attempt to infer co-factor TFs—a conceptual advance that addresses a genuine gap in earlier methods.

Wang et al. adapted their previously developed method, Taiji, to predict cellular reprogramming protocols, naming the new approach Taiji-reprogram^17,18^. This method integrates transcriptomic and epigenomic data to generate cell-type-specific GRNs and uses the personalized PageRank algorithm to evaluate the influence of each TF per cell type.

Active regulatory regions are identified from ATAC-seq, DNase-seq, or H3K27ac ChIP-seq peaks and linked to their interacting genes using EpiTensor. These regions are scanned for TF binding sites using motifs from the CIS-BP database. In the resulting GRN, the node weights corresponds to gene expression level, while edge weights are proportional to the expression level of the regulating TF.

After calculating a PageRank score for each TF in every cell type, Taiji-reprogram calculates the ratio between the TF’s score in the target and starting cell types. TFs with the highest absolute ratio values are selected as candidates. For all combinations of three candidate TFs, the product of their ratio scores is calculated and top TFs are selected based on their frequency in the highest-ranking combinations.

Adapting the PageRank algorithm for identifying TFs in reprogramming is an intriguing concept; though it may not be optimal for identifying master regulators. The PageRank algorithm favors TFs that are well-connected in both directions, meaning those with significant inputs (being regulated) and outputs (regulating others). Master regulators relevant to transdifferentiation, however, should regulate many downstream genes, while being minimally regulated themselves ensuring robust and autonomous activity. Incorporating alternative centrality measures could improve identification of such TFs: out-degree centrality would highlight TFs with strong downstream influence, while betweenness centrality might reveal TFs acting as bridges between regulatory modules.

Xu et al. proposed ANalysis Algorithm for Networks Specified by Enhancers (ANANSE), based on their observation that tissue-specific TFs predominantly bind to enhancers^19^. Using ChIP-seq data from 296 TFs in the ReMap database^20^ and the Human Protein Atlas^21^ classification of tissue-specific TFs, they demonstrated that cell type-specific TFs primarily bind to putative enhancer regions.

ANANSE operates through three interconnected modules. The first predicts the binding probability of TFs with known motifs in enhancer regions by integrating TF motif scores, enhancer activity (measured by ATAC-seq and/or H3K27ac ChIP-seq), and optionally, average TF ChIP-seq signal from ReMap. The second module calculates a TF-gene binding score by aggregating all predicted TF binding probabilities within 100 kb of a gene’s transcription start site (TSS), weighted by their distance from the TSS. This score, combined with the TF’s genome-wide activity derived from ATAC-seq and/or H3K27ac ChIP-seq, and the expression levels of both the TF and its target gene, yields an interaction score (the mean of these four scaled values). GRNs for both the source and target cell types are inferred from these interaction scores. The third module, computes an influence score for each TF, estimating its contribution to expression differences between the two cell types. This score is derived from the differential GRN and considers the log2 fold changes in gene expression, the TF-gene interaction score, and the network distance between the TF and its target genes.

ANANSE incorporates conformational information in the form of enhancer-promoter links and integrates various types of data, yet it lacks the consideration of TF cooperativity and further factors that will be discussed in the following chapter.

### Adaptation of alternative computational tools

In addition to methods specifically designed to find TFs for direct reprogramming, a wide range of computational tools has been proposed for identifying reprogramming cocktails. For example, tools developed for TF activity inference, defined by the regulatory influence a TF exerts on its target genes^22^, can be applied to this task. While many of these tools natively support detecting TFs with differential activity between two cell types, others may require adaptation to fit the specific requirements of cell reprogramming studies. However, these tools typically have limited capabilities in accounting for combinatorial TF effects. They do not consider factors such as TF combinations, synergies, and activation order.

Similarly, methods designed to model perturbation effects can theoretically assist in identifying suitable TF candidates by predicting perturbations that shift a donor cell’s state toward the target cell type^23^. Nevertheless, perturbation methods require combinatorial testing of TF sets, which can be computationally highly intensive. For instance, testing all possible combinations of four TFs would involve approximately six trillion calculations—a scale that is only feasible with substantial computational resources and efficiently designed algorithms. Furthermore, perturbation predictions show only local effects and it remains unclear if this is the most efficient path to the target cell type.

To identify intermediate states and barriers in transdifferentiation procedures, several tools show potential and may be especially useful for refining existing transdifferentiation protocols.

CELLoGeNe uses Boolean GRNs to map all possible states onto an energy landscape, enabling identification of stable and metastable cell states along with their associated TFs^24^. Using energy landscape simulations with weighted random walks (analogous to marbles rolling downhill), the method predicts where cells end up upon perturbation and identifies roadblock states where cells become trapped during reprogramming. However, the model is restricted to core GRNs (networks with >30 TFs become intractable), and transiently expressed TFs that are absent from both donor and target states will be missed if not explicitly added based on prior knowledge or experimental data.

A similar approach is MuTrans, which constructs random walk transition probability matrices from single-cell data and maps them onto an energy landscape where stable states reside in attractor basins and transitions occur through saddle points^25^. The method calculates a Transition Cell Score to distinguish stable from transitioning cells and identifies transition-driver genes whose expression correlates with the transition process. The method has the advantage that it can infer from the whole single-cell transcriptome space; but it does not provide causal information.

Another set of tools holding potential for direct reprogramming research are CARDAMOM and HARISSA^26,27^. Both are built on the same mechanistic mathematical framework that reconstructs gene regulatory networks and provides quantitative, interpretable parameters rather than mere correlation scores. Using time-series data, CARDAMOM infers developmental landscapes where cells occupy metastable states during differentiation. While CARDAMOM is computationally efficient and best suited for networks of 5–20 genes (with decreasing accuracy at larger scales), HARISSA enables accurate simulation-based perturbation modeling^28^. This integrated inference-simulation framework could be applied to existing transdifferentiation protocols to computationally test interventions that improve conversion efficiency before experimental validation, while gaining a mechanistic understanding of the driving forces.

Another tool that uses time-course scRNA-seq data is Waddington-OT (Optimal Transport) which tries to solve the optimal transport problem while explicitly modeling cell proliferation and death through unbalanced transport theory^29^. This enables developmental history tracing and time-lagged regulatory modeling that distinguishes immediate regulators from pioneer factors acting days in advance. Also, the method can incorporate paracrine signaling analysis. The explicit modeling differential growth and death rates, which can be critical in low-efficiency reprogramming processes and the ability to also incorporate signaling molecule information makes this model a very interesting candidate for the application in transdifferentiation research. Particularly noteworthy is that the authors performed experimental validation in fibroblast-to-iPSC reprogramming and could confirm two predictions: adding TF Obox6 to the OSKM protocol increased efficiency 2-fold, while supplementing with cytokine GDF9, the molecule with highest paracrine interaction score, yielded 4–5-fold improvement.

While these methods are valuable for optimizing existing protocols when intermediate state data is available, limitations remain for discovering completely novel reprogramming strategies. These include the need for intermediate state characterization, computational complexity in modeling all TF combinations, and difficulty detecting transiently expressed TFs absent from donor and target populations. Nevertheless, their ability to identify roadblocks, quantify barriers, and predict key regulators makes them powerful tools for rational transdifferentiation optimization.

### Common limitations of all approaches

While we have outlined specific limitations of the currently available computational approaches for guiding reprogramming, these methods face shared challenges that impede their performance. The first set of challenges pertains to factors that, in principle, could already be addressed through algorithmic advancements, such as integrating information about TF cofactors. The second set of challenges, however, arises from data constraints: some data, like insights from spatial omics, are only now becoming accessible, while other types, such as proteomics data on post-translational modifications of TFs, remain insufficiently available.

While the human genome encodes approximately 1600 TFs, alternative splicing and protein processing events generate more than 3500 distinct active forms of these proteins^30^. Isoforms of the same TF can exhibit significant functional diversity, varying in their DNA-binding specificity, subcellular localization, interactions with cofactors and other transcriptional regulators, and even their transcriptional activity. For instance, the full-length p53 protein promotes apoptosis and suppresses tumor growth. In contrast, its truncated isoform, Δ40p53, contributes to cell survival, proliferation, and potentially tumor progression by acting as a dominant-negative regulator of the full-length protein^31^.

In a study by Joung et al., the functional impact of TF isoforms on direct reprogramming was systematically analyzed using an experimental screening approach that assessed their influence on the differentiation of human embryonic stem cells^30^. The results revealed that there were often drastic differences in differentiation efficiency between isoforms of the same TF gene. All current reprogramming tools treat TFs as a singular entity and do not incorporate any available data on isoforms.

DNA methylation, the addition of a methyl group to a cytosine base followed by a guanine (CpG dinucleotide), is a key factor in regulating cell-type-specific transcription patterns. This modification influences transcription by altering the binding affinity of TFs at regulatory regions and modulates chromatin structure by recruiting or inhibiting histone-modifying enzymes. While promoter methylation is typically associated with transcriptional repression, the effects of methylation can vary depending on genomic context and the specific TFs involved^32^.

The establishment of new methylation patterns is orchestrated by the DNA methyltransferases DNMT3A and DNMT3B. Conversely, DNA demethylation is predominantly facilitated by the Ten-Eleven Translocation (TET) family of enzymes^32^. These enzymes can be precisely guided to specific genomic loci with the help of TFs and long noncoding RNAs (lncRNAs)^33^. The influence of DNA methylation on TF binding varies depending on the transcription factor and the methylation status of the binding site. While some TFs exhibit reduced binding affinity to methylated CpG regions, some demonstrate an enhanced preference for methylated sequences, and other TFs remain unaffected by the methylation status^34^.

None of the current reprogramming tools account for target sites’ methylation status. Overexpressed TFs, although they might be crucial for establishing target transcriptional patterns, may be unable to bind their target genes if key regulatory regions are methylated. Conversely, introduced TFs may inappropriately bind to methylated sites not bound in the native target cell, leading to aberrant gene expression. Current reprogramming cocktails also struggle to silence active genes from the starting population that are natively inactive in the target cell, which leads to incomplete reprogramming and the emergence of hybrid cell types^9^. Integrating knowledge of DNA methylation patterns will be essential for future approaches to tackle these obstacles. The tools must infer if DNA methylation-modulating factors should be part of TF cocktails and factors that can alter DNA methylation at certain sites. This could be the addition of lncRNAs known to target and silence specific regions^35^ or epigenetic editing, which employs CRISPR-based tools, that can precisely modulate methylation at specific genomic loci^36^. Additionally, it would be beneficial to validate whether broad-spectrum DNMT inhibitors, such as 5-azacytidine (5-aza) and RG108^37^, both of which have been shown to enhance certain reprogramming protocols, could tangible benefits in this context.

Transcription co-factors (TcoFs) interact with TFs and perform diverse functions, including modulating TF–DNA binding, chromatin modification, bridging regulatory complexes, and signal transduction^38^. Based on our current knowledge, there are around 1000 putative human TcoFs that are expressed in a cell-type-specific manner and play a crucial role in fine-tuning transcriptional activity by integrating multiple signaling pathways. It was suggested that, on average, a human TF interacts with around 6 TcoFs, although this number may vary by cell type or TF family^39^.

Despite their importance, current approaches neglect TcoFs and their role in guided differentiation. It will be essential that future tools go beyond TFs alone and also suggest TcoFs that are necessary for the TFs to fully exert their regulatory potential.

TFs can engage in either direct or indirect competitive binding at their DNA binding sites. Direct competition occurs when TFs compete for the same binding site, while indirect competition is mediated by TFs recruiting cofactors that either enhance or inhibit the binding of other TFs. This competition can result in differential regulation of the gene controlled by the binding site.

Paralogous TFs, which arise from the same ancestral gene, often compete with each other^40^. While these proteins typically share similar DNA-binding attributes, they can differ significantly in their functional outcomes. An example are CTCF and BORIS, two paralogous proteins sharing nearly identical DNA-binding domains, enabling them to bind the same DNA sequences. While CTCF is ubiquitously expressed in somatic cells, BORIS is restricted to male germ-line cell where it replaces CTCF and establishes a cell-type specific expression pattern^41^.

Studies have shown that in humans, across 37 tissue types^42^, 43 out of 58 TF families with more than one paralog have at least two family members coexpressed in at least one tissue type. This coexpression highlights the potential for competitive interactions between paralogous TFs in regulating gene expression.

Current approaches to cellular reprogramming and TF-based interventions largely overlook the possibility that the expression of a competitive TF in the target cell may interfere with the activity of the introduced TF. Future strategies should address this limitation by identifying competitive TFs that may need to be silenced alongside those that require overexpression. Incorporating this consideration could significantly improve the efficacy and precision of TF-mediated cellular reprogramming and gene regulation protocols.

Many cell types express specific miRNAs that suppress the translation of lineage-inappropriate transcripts, ensuring proper cell identity and function^43^. In some reprogramming protocols, overexpressed TFs have been shown to achieve effective lineage conversion only when specific miRNAs are co-expressed^44^. Despite this, current computational tools for cellular reprogramming overlook the critical role of miRNAs in regulating lineage-specific gene expression. Incorporating miRNAs (or TFs that activate these miRNAs) that specifically silence donor cell population-specific genes into these tools could significantly enhance their accuracy and usability, enabling more efficient and faithful reprogramming.

Current algorithms have primarily been designed to analyze sequencing data from microarray or bulk sequencing experiments, which generally come from non-homogeneous tissues or mixed cell populations. The advent of single-cell sequencing, however, has enabled measurements from pure cell populations and uncovering details about their varying cell states and expression levels along differentiation trajectories. Moreover, integrating bulk RNA-seq data with single-cell data presents an opportunity to leverage the vast repository of existing bulk experiments. By employing advanced machine learning approaches such as lifelong learning^45^, which are now being explored for GRN inference^46^, future methods could bridge the gap between these data types and enhance predictive power.

An important consideration is the transient nature of certain TFs during cellular differentiation. These TFs are expressed only temporarily, fulfilling specific roles before being silenced^47^. Traditional single-cell RNA sequencing provides only static snapshots of gene expression. However, new methods like single-cell metabolically labeled new RNA tagging sequencing (scNT-seq) enables the simultaneous profiling of newly transcribed and pre-existing mRNAs within individual cells^48^. By incorporating temporal dynamics into computational frameworks, future tools could propose multi-step transdifferentiation protocols tailored to these dynamic changes rather than relying on a single cocktail of TFs.

By providing a deeper understanding of the tissue microenvironment spatial omics data offer another avenue that might greatly improve future reprogramming procedures. Spatially resolved data can reveal critical interactions between neighboring cells and the extracellular matrix, highlighting the importance of cell–cell communication and microenvironmental cues in controlling cell-type-specific transcriptional programs.

Researchers might identify ligands, receptors, and mechanical signals influencing cellular behavior and differentiation. For instance, paracrine signaling from neighboring cells often plays a key role in regulating target cell fate, while physical interactions with the extracellular matrix can affect chromatin organization and gene expression^49^.

A common limitation of all approaches is their reliance on transcriptional data, which only moderately correlates with protein levels^50^. As proteins—not RNA—are the primary executors of cellular functions, this presents a significant hurdle. This disconnect is further compounded by the fact that proteins, particularly TFs, exhibit diverse functionalities due to post-translational modifications (PTMs). PTMs introduce substantial differences between a TF and its modified counterparts, often altering highly important properties. For example, modifications like phosphorylation can influence a TF’s activity, subcellular localization, DNA-binding ability, and interactions with cofactors or other TFs^51^.

**Fig. 2:**
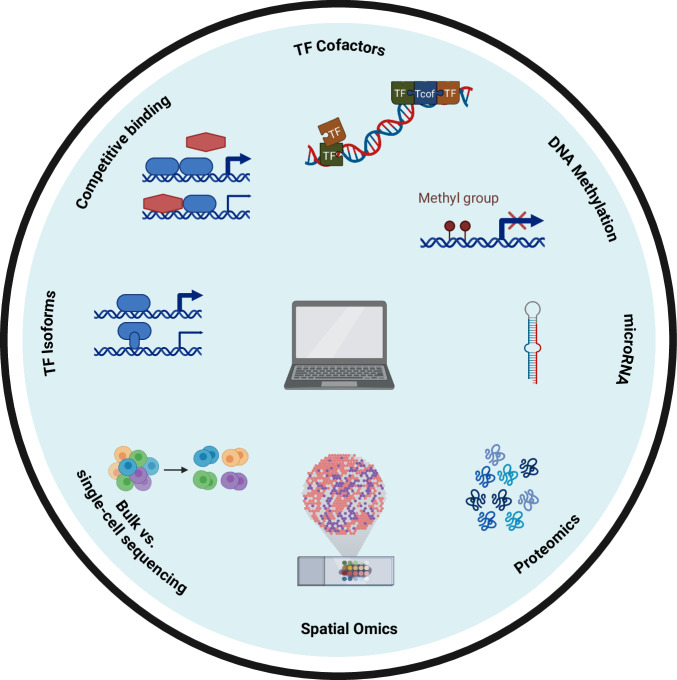
Limitations of current computational procedures. Current tools do not incorporate existing knowledge about TF isoforms, competitive binding of TF, TF cofactors, DNA methylation at TFBS and the regulatory activity of microRNA. Further, emerging profiling technologies like spatial omics, proteomics and single cell technologies are not yet made use of. Figure created with BioRender.com.

While we currently lack the ability to produce high-throughput datasets that capture PTMs of all TFs in a cell-type-specific manner, it is foreseeable that, with current progress in single-cell proteomics, this will become possible in the future. At that point, computational tools must incorporate this additional layer of information (Fig. 2).

### A guide to method choice

Despite the limitations of existing approaches, it is worthwhile to explore concurrent strategies for dynamic reprogramming. While it may be tempting to assume that newer methods, which utilize a broader range of data types, are inherently superior to older ones, we currently lack the data to substantiate such claims. While many of the described algorithms conducted benchmarking studies in their initial publications, these evaluations have several limitations.

Tools like Mogrify and D’Alessio et al.’s approach do not provide accessible code to easily rerun their analyses with new data. As a result, comparisons with other tools often rely solely on predictions reported in the original publications. These predictions were based on datasets available at the time of publication, hence it is unclear whether observed performance differences stem from algorithmic limitations or outdated data. Given that data quality and quantity are critical factors in generating reliable predictions, it remains unclear how these tools would perform on more recent, higher-quality datasets.

Another issue is that benchmarking studies frequently compare algorithms against a limited set of competing methods (e.g., ANANSE vs. CellNet). This selective comparison leaves uncertainty about whether omitted algorithms were excluded due to superior performance or other reasons.

When it comes to computational methods that were repurposed, such as TF activity methods to find reprogramming factors, no independent high-quality benchmark exists.

Hammelman et al. tested nine different computational approaches not originally designed for the inference of reprogramming protocols^52^. The methods they tested relied on either gene expression (EBSeq, CellNet), chromatin accessibility (DREME, AME, Homer, KMAC, diffTF, DeepAccess), or a combination of the two (GarNet). Here, they showed that motif enrichment via AME^53^ on differentially open chromatin position was superior compared to the other methods.

However, while previous benchmarks focused on protocols for the reprogramming of human cells, Hammelman et al. exclusively analyzed murine cells. Furthermore, they clustered all TF motifs into 107 mouse transcription factor motifs and considered a prediction successful if the predicted clustered motif included the correct TF. This ultimately limits the usefulness of their study.

### DiReG: an interactive webtool for the discovery of transcription factors for direct reprogramming

Given the limitations of current reprogramming protocols, we have developed a web application to assist experimental scientists in identifying novel reprogramming cocktails. We named the DiReG: The Direct Reprogramming Guide.

DiReG enables users to query existing reprogramming literature through both PaperQA (offering higher accuracy but greater computational cost) and a custom Retrieval-Augmented Generation (RAG) pipeline that provides faster, more economical analysis with integrated citation tracking.

**Fig. 3:**
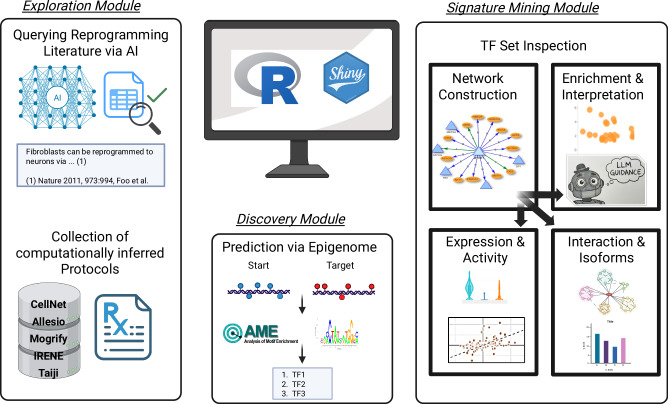
Overview of the functionality of the web application. Three distinct modules allow the user to explore existing knowledge (Exploration Modulation), make a new prediction (Discovery Module) or inspect a set of TF for their potential to induce transdifferentiation (Signature Mining Module). Figure created with BioRender.com.

Additionally, DiReG allows users to explore predictions from traditional reprogramming approaches, such as CellNet or Mogrify, and incorporates AME to infer TF sets using epigenomic data. Finally, at its core, the application provides a suite of functions to evaluate the potential of different TF sets, whether derived from the user’s expert knowledge or generated through the aforementioned approaches (Fig. 3).

### Exploration module: querying validated and inferred predictions

#### Literature querying via retrieval-augmented generation

We have compiled a collection of 360 freely available publications related to direct reprogramming and integrated both a language model agent called PaperQA^54^ and a custom-made RAG to enable users to ask topic-specific questions. The literature database is designed for seamless updates through our automated pipeline, which can be run periodically to incorporate newly published research without manual intervention.

Our systematic approach utilizes the NCBI E-utilities API with a standardized search query targeting direct reprogramming publications while excluding reviews and other non-primary content. The automated pipeline performs title-based filtration using a LLM to classify publications, retaining only those relevant to cell biology. This standardized, programmatic methodology allows for regular database refreshes—simply rerunning the pipeline captures new publications that match our criteria, ensuring the system remains current with minimal maintenance.

PaperQA provides high-accuracy responses that surpass human performance in scientific literature research, working with both closed-source and open-source language models. Users can register with their OpenAI account to query the literature using their preferred model. While a single PaperQA request costs several cents and may take minutes to process, we also implemented a complementary lightweight RAG system that delivers responses in under 30 s at less than one cent per query, enabling cost-effective initial literature exploration. This dual-system approach provides both depth and efficiency, helping users quickly retrieve information about existing protocols and experimental details while circumventing time-consuming literature searches.

#### Computationally inferred reprogramming TF sets

Many existing approaches neither provide an easy way to run calculations from scratch nor offer a convenient method to query precomputed results, even when such data is available. In many cases, the inferred data is buried in supplementary materials, making it difficult for users to access and utilize.

To address this, we present a unified resource that allows users to access results from CellNet, D’Alessio et al., Mogrify, IRENE, and Taiji-Reprogram. Users can filter by the method of interest and select their starting and target cell types from a dropdown menu. By clicking on the predicted TFs, the selected TF set is seamlessly imported into the Mining Module, where users can further explore and analyze predictions using various tools, as described in the Hypothesis-driven Inspection of TF Candidates section.

### Discovery module: inference of TF candidates using AME

AME, a part of the MEME suite, was initially developed to identify motifs that are relatively enriched in a set of sequences compared to control sequences^53^. Hammelman et al. demonstrated that this method outperforms other approaches repurposed for finding reprogramming TFs. While we cannot directly compare its performance with the other methods discussed, we decided to include it due to its simplicity, minimal data requirements (open chromatin data), and efficient runtime.

Within the app, users can upload data—such as ATAC-seq for a starting and target cell type—select a set of motifs and an appropriate reference, and run the method using shuffled or background-matched sequences as a control.

### Signature mining module: hypothesis-driven inspection of TF candidates

Many researchers have an intuitive sense of which TFs might be suitable for generating a specific cell type. We provide tools designed to validate and explore these TF candidates to support this intuition. The platform allows users to visualize all potential downstream targets of their candidate TFs within an interactive network based on the CollecTRI database, providing an intuitive understanding of their regulatory influence.

Additionally, users can perform both Overrepresentation Analysis (ORA)^55^ and Gene Set Enrichment Analysis (GSEA)^56^ on the targets of these TFs to determine whether they show enrichment specific to the desired organ or tissue. The results of these analyses are accompanied by guidance from an LLM to help interpret the findings effectively.

The platform further enables users to explore the expression levels of their candidate TFs in both donor and target tissues, as well as their differential activity, offering another layer of validation for their relevance to the reprogramming process. Finally, the tool provides insights into whether different isoforms of the TFs exhibit varying reprogramming potentials at the iPSC level, which may hint at their functional roles in the target cell type.

This comprehensive set of features empowers researchers to refine and validate their TF choices, aiding in the design of effective reprogramming protocols. In the following, we give a more detailed outline of the module and its sub-tools.

#### Input TF specific gene regulatory network

After the user inputs a set of TFs, a network is generated that visualizes these TFs and their downstream targets. This TF network, derived from the CollecTRI network^57^, is a comprehensive tool for exploring genes regulated by specific TFs. The CollecTRI network is curated from diverse sources of evidence and provides an extensive collection of transcription factors and their transcriptional targets.

**Fig. 4:**
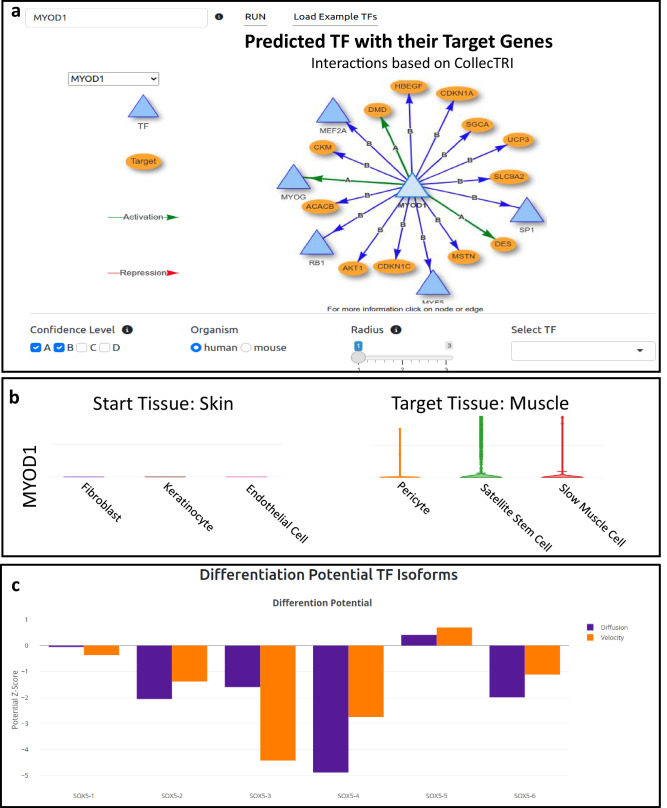
Interactive validation features within the Signature Mining Module. **a** The Gene Regulatory Network (GRN) interface, shown here using *MYOD1* as a representative example. The view visualizes the user-selected TF and its predicted downstream targets based on the CollecTRI database. **b** Expression profiling interface displaying violin plots for the selected candidate (e.g., *MYOD1*). This feature allows users to assess expression differences between defined source tissues (left, e.g., Skin) and target tissues (right, e.g., Muscle). **c** Isoform potential analysis, illustrating the differential differentiation capacity of various TF isoforms (shown here for *SOX5*) on hPSCs to guide specific isoform selection.

The network is highly customizable, allowing users to expand it by including not only the primary targets of input TFs but also the targets of those induced factors, thereby creating a more comprehensive view of regulatory relationships. Users can set confidence thresholds to filter interactions based on reliability, ranging from curated high-confidence interactions to those with lower confidence. For large networks, users can focus on specific TFs to enhance clarity. To facilitate deeper exploration, users can click on any gene node to be redirected directly to its corresponding PubMed entry for more detailed information (Fig. 4a).

To further enrich the analysis, DiReG offers a suite of downstream tools for characterizing the generated network, which are explained in the following section.

#### Enrichment analysis

Once the GRN is generated, the next critical question is how closely the network resembles the target cell. Specifically, do the genes activated by the input TFs show enrichment in the target cell, and are the repressed genes effectively silenced in the target cell type? A common approach to validating a GRN is enrichment analysis. To facilitate this, we have implemented two complementary methods within the app: Overrepresentation Analysis (ORA)^55^ and Gene Set Enrichment Analysis (GSEA)^56^.

**Fig. 5:**
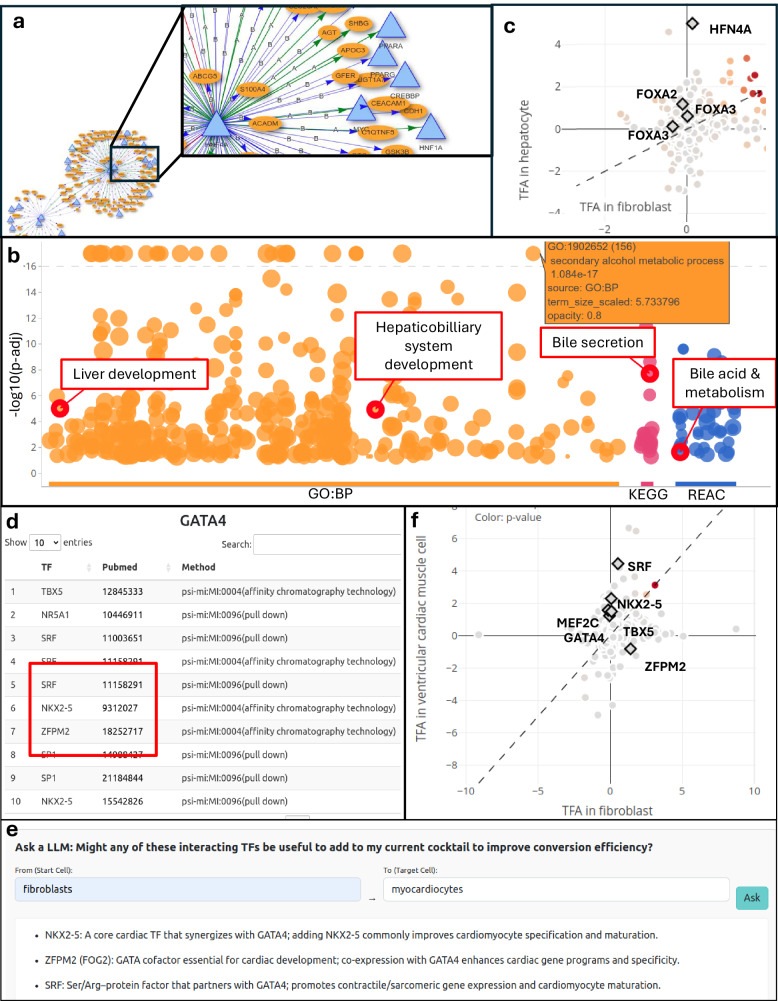
Validating and optimizing transcription factor combinations using DiReG. **a** Regulatory network visualization generated for the HNF4A and FOXA TF combination, highlighting the downstream activation of additional liver-related regulators such as HNF1A, PPARG, and PPARA. **b** Overrepresentation analysis of the generated network showing significant enrichment for liver-specific processes across GO: BP, KEGG, and REAC databases. **c** TFA scatter plot comparing hepatocytes versus fibroblasts where HNF4A, FOXA1 and FOXA2 exhibit hepatocyte-specific activity. **d** TF-TF interaction table for the cardiac factor GATA4. Red box highlights identified interactors—SRF, NKX2-5, and ZFPM2—validated by literature. **e** The integrated LLM support module suggesting these interacting factors (NKX2-5, ZFPM2, SRF) as candidates to improve the conversion efficiency of the GMT cocktail for cardiomyocyte reprogramming. **f** TFA scatter plot comparing ventricular cardiac muscle cells versus fibroblasts, demonstrating the cardiac-specific activity of the GMT cocktail and the proposed candidates.

ORA uses Fisher’s exact test to detect overrepresented gene sets by assessing the proportion of genes in a set that are present in the network. Gene sets with an adjusted *p*-value below 0.05 are considered significant. These results are visualized in an interactive dot plot, where the y-axis represents the adjusted *p*-value. GSEA ranks genes based on factors such as their proximity to input transcription factors, confidence scores, and regulatory modes, calculating enrichment scores for predefined gene sets. Significant gene sets with the highest enrichment scores are displayed in a classic dot plot, where the x-axis represents the normalized enrichment score. Figure 5b demonstrates how this analysis identifies liver-specific processes in a hepatocyte reprogramming scenario.

To enhance the interpretability of significant gene sets, users can query a LLM directly within the app to assess potential relevance to specific cell types or biological contexts. This feature provides immediate insights, helping users contextualize their findings more effectively. As in the literature querying section, a user can register with his or her OpenAI account and query using a preferred model.

ORA and GSEA results are presented in an interactive, sortable table, allowing users to filter by specific genes, criteria, or significance thresholds. Additionally, all results can be downloaded as CSV files, enabling further analysis in external tools or frameworks.

#### Tissue and cell type specific expression

To further evaluate the suitability of a set of TFs for reprogramming, it is essential to determine whether a TF of interest is expressed in the tissue of interest and whether there are significant differences in its expression levels between the target and donor cell types. DiReG provides a comprehensive visualization of expression levels for the input TFs across all cell types within selected tissues to support this analysis.

For human cell types, the app leverages single-cell RNA-sequencing data from the Tabula Sapiens project^58^, while for murine cell types, it utilizes data from Tabula Muris Senis^59,60^. Expression patterns are displayed as interactive violin plots for each transcription factor, allowing researchers to compare expression distributions across all cell types within a tissue and aiding in selecting TFs with the highest potential for successful reprogramming (see Fig. 4b for a representative view using MYOD1).

#### TF interactions

For DiReG, we expanded and integrated the Transcription Factor Cofactor Database (TcoF-DB). While the original TcoF-DB^39^ provided valuable TF-TcoF interaction data, it had not been updated since 2016. Following the same methodological principles, we have generated a comprehensive update that increases coverage by expanding human transcription factor cofactors from 958 to 1379 and murine cofactors from 418 to 666.

The interface offers interactive exploration of potential cofactors for each input TF displaying the level of evidence supporting each interaction. Users can select any cofactor to immediately visualize its expression pattern across cell types in their tissue of interest through interactive plots (Fig. 4e).

Additionally, we have implemented a complementary database of TF-TF interactions derived from the same updated protein–protein interaction dataset. This resource allows users to identify potential transcriptional partners that might synergize with their primary factors of interest. Both databases are fully integrated with the expression visualization system, providing seamless navigation between interaction data and cellular expression patterns to inform optimal factor selection for reprogramming cocktails. Figure 5d shows an example of a TF-TF interaction table identifying potential cofactors for GATA4.

#### Isoform potential

Joung et al.^30^ demonstrated that different isoforms of a transcription factor can exhibit varying potentials for reprogramming and cellular differentiation. This information could be highly valuable for researchers focusing on specific isoforms, as certain isoforms may have unique relevance in their donor cell type. To assist in understanding these differences, we present the differential potential of transcription factor isoforms on hPSCs using bar plots, providing a clear visual representation of their relative efficacy (Fig. 4c).

#### TF activity

To evaluate whether transcription factors exhibit differential regulatory activity between donor and target cell types DiReG integrates decoupleR^61^, a framework for network-based inference of transcription factor activity. Activity inference quantifies a transcription factor’s regulatory influence by integrating both its expression and the expression patterns of its downstream target genes.

The application processes bulk RNA-sequencing data from GTEx and single-cell data from Tabula Sapiens for human samples or Tabula Muris Senis for murine models to calculate regulatory activity scores for TFs covered by the CollecTRI database. Multiple statistical approaches are applied simultaneously, with the consensus mode providing robust activity estimates. This approach may identify TFs that function as key regulatory nodes in either donor or target cell types, even when their expression differences alone may be subtle.

The results are visualized as an interactive dot plot, where TF activity in the target cell type is plotted on the y-axis and activity in the donor cell type is plotted on the x-axis. TFs that exhibit low activity in the donor cell type but high activity in the target cell type are highlighted as ideal candidates for inclusion in reprogramming cocktails. Input TFs are distinctly marked as diamonds with bold outlines, and a color gradient indicates the statistical significance (*p*-value) of their activity differences. Figure 5c, f show the distinct activity separation achieved by master regulators in hepatocytes and cardiomyocytes, respectively.

Users can download the complete set of results to support further analysis, enabling detailed exploration and integration into other analytical workflows.

### Application examples: validating transcription factor combinations using DiReG

To demonstrate the practical utility of the signature mining module, we present two case studies that illustrate how DiReG can validate and optimize transcription factor combinations for direct reprogramming. First, we examine the validation of HNF4A in combination with FOXA genes as drivers of hepatocyte generation. Second, we explore how DiReG can be used to improve an existing reprogramming cocktail, specifically the GATA4, MEF2C, TBX5 set, which successfully generates cardiomyocytes in murine cells but exhibits limited efficiency in human cells.

#### Example 1: Validation of HNF4A-FOXA combinations for hepatocyte reprogramming

HNF4A and members of the FOXA family (FOXA1, FOXA2, FOXA3) are well-established master regulators of hepatocyte development and identity. Recent work has demonstrated that any combination of HNF4A with any single FOXA transcription factor is sufficient for hepatocyte induction^62^.

Using DiReG’s signature mining module, we analyzed these factor combinations by generating regulatory networks at high confidence levels (categories A and B). Each combination produced networks containing 110–140 genes. Visual inspection of these networks (Fig. 5a) immediately revealed downstream activation of additional liver-related transcription factors, including HNF1A, PPARG, and PPARA, downstream of the input factor HNF4A.

Overrepresentation analysis of the generated networks (Fig. 5b) demonstrated clear enrichment of liver- and hepatocyte-related biological processes. To validate these findings, we employed the integrated LLM support function, which provided the following assessment of hepatocyte specificity (Box 1).

To verify that the LLM analysis was not merely confirming user expectations, we tested whether the enriched gene sets showed specificity for an unrelated cell type, cardiomyocytes. The LLM correctly identified the absence of cardiac signatures, concluding: “The enriched gene sets do not show specificity for cardiomyocytes. Strong liver/intestine-associated processes appear instead.” This negative control validates the robustness of the analytical approach.

Expression analysis revealed that each transcription factor exhibited moderate to high hepatocyte-specific expression, while expression in fibroblasts was low or absent, providing a clear rationale for overexpressing this factor set in fibroblast-to-hepatocyte conversions (Supplementary Fig. S1).

Furthermore, transcription factor activity analysis (Fig. 5c) demonstrated that all factors except FOXA3 showed strong hepatocyte-specific regulatory activity, indicating that these factors are natively active in hepatocytes and absent or inactive in fibroblasts. This comprehensive analysis demonstrates DiReG’s capability to provide multi-layered evidence supporting strong transdifferentiation transcription factor candidates.

#### Example 2: Optimization of the GMT cocktail for human cardiac reprogramming

The GMT cocktail (GATA4, MEF2C, TBX5) efficiently reprograms murine fibroblasts into cardiomyocytes but exhibits insufficient conversion rates in human cells^63^. We utilized DiReG to investigate potential improvements to this factor combination.

Initial analysis of the GMT set through enrichment analysis and subsequent LLM evaluation revealed an enrichment of cardiac-related gene sets. The LLM provided the following assessment (Box 2).

This cardiac signature could be interpreted either as a false positive or, more likely, as confirmation that these factors provide a solid foundation for cardiac reprogramming that requires optimization.

As a negative control, we queried whether these gene sets showed hepatocyte specificity. The LLM correctly identified the lack of hepatic signatures, stating: *“No. The enriched sets you listed do not show hepatocyte-specific signals. They are heavily weighted toward cardiac/skeletal muscle development.”* This further validated the specificity of the analytical approach.

To identify potential factors that could improve conversion efficiency in human cells, we examined the TF-TF interaction analysis within DiReG (Fig. 5d). This revealed several cardiac-relevant factors that interact with the GMT set, including SRF, NKX2-5, and ZFPM2, all of which interact with GATA4 and are established regulators of cardiac development.

We utilized DiReG’s integrated LLM function (Fig. 5e) to automatically evaluate the set of interacting transcription factors for their relevance to cardiac cell types. In most analyses, the LLM independently identified these three factors as high-priority candidates. Literature validation supports the potential of these additions:

ZFPM2 (also known as FOG2) is a cofactor for GATA4 that does not bind DNA directly but recruits GATA4 to cardiac-specific enhancers while preventing binding to non-cardiac targets such as liver or gut enhancers. ZFPM2 was included in a seven-factor cocktail that demonstrated efficient reprogramming of human cells^63^.

SRF has been reported to enhance reprogramming efficiency when added together with MYOCD to the GMT cocktail in human fibroblasts^64^.

NKX2-5 is an essential transcription factor for early heart formation and has been shown to improve reprogramming in various murine cocktails. However, its role in human cardiac reprogramming is more complex: while it facilitates the initiation of cardiac reprogramming, it may block complete maturation to fully functional cardiomyocytes^63^.

Regarding transcription factor cofactors, the analysis identified several potential cofactors for the GMT factors within the database. However, notably, BAF60C (SMARCD3)—a chromatin remodeling cofactor that has been experimentally validated to improve cardiac reprogramming protocols^65^ and represents, to our knowledge, the only well-characterized cofactor known to enhance cardiac reprogramming efficiency—was not identified by the analysis.

Finally, transcription factor activity analysis confirmed that both the original GMT set and two of the newly identified candidates (SRF and NKX2-5) exhibit distinct regulatory activity in cardiac muscle cells compared to fibroblasts (Fig. 5f), further supporting their role in driving cardiac identity.

These case studies demonstrate how DiReG’s signature mining module provides researchers with a systematic framework to validate existing transcription factor combinations and identify optimization strategies for reprogramming protocols. By integrating network analysis, enrichment testing, LLM-assisted interpretation, expression profiling, activity inference, and interaction mapping, DiReG enables comprehensive evaluation of candidate factors and rational design of improved reprogramming cocktails.

Box 1 LLM-assisted validation of HNF4A-FOXA hepatocyte reprogramming The enrichment strongly supports hepatocyte- or liver-like metabolism, driven by classic hepatic functions such as xenobiotic processing, conjugation, and central liver metabolic pathways. Specific liver-centric terms in your list include:

- *Liver-specific development pathways: liver development, hepaticobiliary system development, tissue development, and regulation of liver/organ development.*
- *Hepatic metabolic functions: bile secretion, bile acid and bile salt transport, cholesterol transport, HDL/LDL remodeling and clearance, lipid digestion and absorption, and cholesterol homeostasis.*
- *Xenobiotic metabolism (a hallmark of hepatocytes): metabolism of xenobiotics by cytochrome P450, drug metabolism pathways, and metabolism of vitamins and cofactors.*
- *Hepatic signaling pathways: PPAR signaling, steroid biosynthetic processes, regulation of lipid metabolism by PPARα, and steroid metabolism.*
- *Additional liver-associated processes: retinoid metabolism and transport, plasma lipoprotein particle assembly and clearance.*

Box 2: Diagnostic optimization of the GMT cardiac cocktail *Partial specificity toward cardiomyocytes, with strong cardiomyocyte/heart biology signals but not exclusive*.

*Key cardiomyocyte-relevant signals in your enriched sets:*

- *Cardiac development terms: cardiac muscle tissue development, cardiac muscle cell differentiation, heart development, heart morphogenesis, cardiac chamber morphogenesis and development.*
- *Cardiac structure formation: cardiac ventricle development, atrioventricular valve development, atrial and ventricular septum development.*
- *Cardiac function: cardiocyte differentiation, cardiac muscle contraction, regulation of heart contraction, cardiac conduction system development.*

*Related processes: blood vessel development, angiogenesis, circulatory system processes, and striated muscle differentiation and contraction.*

*While some enriched terms were broad developmental processes common across multiple lineages (e.g., MAPK/PI3K-Akt signaling, regulation of multicellular organismal development), the overall enrichment pattern strongly supported cardiac muscle lineage specification*.

## Discussion

Since the seminal discovery by Davis et al. (1987) that showed that one cell type can be directly converted into another, much progress has been made, and new protocols for direct programming have been discovered. However, these discoveries were coupled with tremendous efforts and work to painstakingly test all different kinds of TF combinations. Advancements in the generation of high-throughput datasets covering different modalities allowed, in the past decade, a diverse array of computational tools to streamline and enhance the identification of TFs essential for lineage conversion.

Our comprehensive analysis of six pivotal computational methods—CellNet, D’Alessio et al.’s core TF framework, Mogrify, IRENE, Taiji-reprogram, and ANANSE—revealed both the progress that was made but various limitations that still persist. We propose a set of factors that future methods must incorporate to leap forward in their useability.

With the identified limitations of existing tools in mind, we developed an interactive web application designed to support researchers in identifying optimal TF sets for direct reprogramming. By incorporating a RAG system, we enable users to efficiently query and extract relevant information from a curated collection of reprogramming publications. By aggregating predictions from CellNet, D’Alessio et al.’s framework, Mogrify, IRENE, and Taiji-reprogram, the web application provides a unified resource that simplifies the comparison and selection of candidate TFs. This integration mitigates the challenge of accessing and interpreting disparate datasets, thereby enhancing usability. The platform’s suite of validation tools allows users to critically assess the functional relevance of their candidate TFs. The application bridges the gap between computational predictions and experimental validation by facilitating the exploration of downstream targets and expression patterns.

In conclusion, by providing a user-friendly platform that integrates existing computational predictions, advanced literature querying, and robust validation tools, we empower researchers to design more effective and precise reprogramming protocols. However, to fully realize the potential of direct reprogramming, future computational tools must address the multifaceted challenges of isoform diversity, epigenetic modifications, TF cofactors, and integrating single-cell, spatial and proteome data.

## Methods

### Functional enrichment analyses of induced networks

Gene overrepresentation analysis was performed using the “gost” function from gprofiler2 (version 0.2.1). The analysis was conducted using nodes from the transcription factor-based directed network as input. Statistical significance was assessed using g:SCS correction method with a threshold of *p* < 0.05.

For gene set enrichment analysis (GSEA), we implemented a network-based gene ranking approach. Our method systematically traversed the transcription factor network up to five hierarchical levels, applying a distance-weighted scoring formula: weight = |mor| × (1/(level × weightFactor)), where |mor| represents the absolute value of edge directionality, level indicates network distance from seed transcription factors, and weightFactor is a scaling parameter. This approach prioritized genes with stronger regulatory connections and proximity to initial transcription factors. The resulting ranked gene list was analyzed using the fgsea package with 500 permutations. Significantly enriched pathways (adjusted *p*-value < 0.05) were identified from multiple MSigDB collections, including Hallmark, Curated (C2), Ontology (C5), and Cell Type Signature (C8) gene sets. The top 10 enriched pathways, ranked by normalized enrichment score (NES), were visualized using interactive plots generated with plotly (version 4.10.1).

### Expression datasets

Human tissue-specific gene expression profiles were obtained from the Genotype-Tissue Expression Portal (https://gtexportal.org/). Specifically, we utilized the “Median gene-level TPM by tissue” dataset from GTEx Analysis Release V8.

Single-cell transcriptomic profiles from diverse human tissues were acquired from the Tabula Sapiens Consortium v2 dataset, which was accessed through figshare (https://figshare.com/articles/dataset/Tabula_Sapiens_v2/27921984).

Murine single-cell transcriptomic data were obtained from the Tabula Muris Senis consortium via figshare (dataset: Processed_files_to_use_with_scanpy_/8273102; https://figshare.com/articles/dataset/Tabula_Muris_Senis_Data_Objects/12654728).

### Protein interaction analysis of transcription factors

Protein–protein interaction data from BioGRID (Version ALL-4.4.241), IntAct (current psimitab 2024-12-05), and Reactome (current release 01/2025) databases was downloaded for both transcription factor cofactor and TF-TF interaction analyses.

To find TF Cofactors data was filtered for species-specific interactions with relevant interaction types (association, physical association, direct interaction, covalent binding). Potential cofactors were identified as non-TF proteins that interact with known transcription factors and further filtered based on Gene Ontology annotations confirming both transcriptional regulation involvement and nuclear localization as described in ref. ^39^.

To find TF-TF Interactions, a more stringent filtering criteria, retaining only direct interactions (MI:0407) and physical associations (MI:0915) between proteins was applied. Species-specific transcription factor identifiers from curated lists were mapped to UniProt accessions using the appropriate organism database (org.Hs.eg.db for human, org.Mm.eg.db for mouse). After standardizing identifiers across data sources, interaction pairs where both partners are confirmed transcription factors were identified.

### Isoform potential

Differentiation potential of transcript isoforms was visualized using RNA velocity and diffusion difference values from ref. ^30^’s Supplementary Table S2 (Sheet B). These values were z-score scaled to enable standardized comparison across isoforms and integrated into the visualization framework to illustrate their relative differentiation capacities.

### TFA calculation

Transcription factor activity (TFA) was calculated using decouplR (version 2.5.3) with a directed regulatory network based on the CollecTRI network where input transcription factors served as source nodes and their downstream targets as destination nodes. Activities were computed using three complementary statistical approaches (ULM, MLM, and WSUM), with final values determined by consensus statistics.

For tissue-level analyses, we used median TPM values from GTEx data. For cell type-specific analyses, we generated pseudobulk samples by aggregating single-cell RNA-sequencing data by tissue and cell type. To ensure robust analysis, only cell types with a minimum of 100 cells per tissue were included.

### Literature mining and analysis using PaperQA and retrieval-augmented generation

To systematically identify primary research articles on direct reprogramming, we queried the NCBI database using the Entrez Programming Utilities (E-utilities) API. The following search query was constructed to target relevant publications while excluding review articles and other non-primary research content:

“direct reprogramming”[Title/Abstract] OR “direct conversion”[Title/Abstract]) NOT (Review[Publication Type] OR Meta-Analysis[Publication Type] OR Systematic Review[Publication Type] OR Editorial[Publication Type] OR Comment[Publication Type] OR Letter[Publication Type]) AND “free full text”[Filter]”.

This query was executed using the esearch.fcgi function with parameters db=pubmed, retmax=2000, and retmode=json to retrieve PubMed and PMC identifiers for freely available articles in JSON format.

For each retrieved identifier, corresponding article titles were extracted using a subsequent E-utilities query. To minimize false positives resulting from our initial search, we implemented a two-stage filtration process:

Title-based assessment using a large language model (GPT-4o), which classified each publication as either “Cell_Biology” or “Other_Field” based on the following standardized prompt:

“Does the following paper title indicate that it speaks about ‘direct reprogramming’ or ‘direct conversion’ in the context of cell biology and cell type conversions, or does it belong to another field like chemistry or physics? Please answer ‘Cell_Biology’ or ‘Other_Field’.\n\nTitle: [ARTICLE TITLE]”. Only publications classified as “Cell_Biology” were retained for subsequent analysis.

The filtered article identifiers were mapped to their corresponding full-text locations on the NCBI FTP server using the reference file “oa_file_list.txt” (version dated 2025-03-08 05:48) from the NCBI PMC open access repository (ftp.ncbi.nlm.nih.gov/pub/pmc/). In total 360 full-text documents (Supplementary Table 1) were then programmatically downloaded.

To enable interactive querying of the retrieved literature via PaperQA within the Shiny application framework we utilized a FastAPI backend that processed user queries against the downloaded full-text articles. Two operational modes were developed: a “fast” mode that examined limited evidence (k = 5) for rapid responses, and a “high_quality” mode that performed comprehensive analysis with expanded evidence retrieval (k = 15).

To generate the custom RAG system, publications were loaded into Python using the UnstructuredPDFLoader module from the LangChain library. For each publication, metadata (title, first author, and DOI) was extracted by feeding the first-page text into a language model (GPT-4o) with a custom-designed prompt optimized to retrieve this information accurately. Following metadata extraction, each document was split into 1000-character chunks with a 200-character overlap using the RecursiveCharacterTextSplitter. The processed data, including both the content chunks and extracted metadata, was embedded and stored in a Chroma vector database. This database was created using embeddings generated by the OpenAI Embedding model to enable similarity-based document retrieval. A multi-query retrieval approach was employed to improve search coverage, generating several variations of user questions for each query to enhance relevance in document retrieval.

The system was implemented as a FastAPI service, providing a consistent interface for integration within our Shiny application. The pipeline incorporates robust error handling with exponential backoff retry mechanisms to manage API limitations. The response generation process utilizes a specialized prompt template instructing the model to cite sources accurately and generates a properly formatted references section.

### AME analysis of differentially accessible regions

For motif enrichment analysis of differentially accessible regions, we implemented a multi-step bioinformatic pipeline within our web application. The pipeline uses transcription factor binding site (TFBS) motifs from either HOCOMOCO (version 11) core mouse/human databases or JASPAR (2024 CORE vertebrates non-redundant) collections, selectable through the user interface.

Input peak files (narrowPeak or broadPeak format) undergo sequential processing: (1) quality filtering to retain only peaks with FDR < 0.05 using custom scripts; (2) identification of differentially accessible regions between conditions using BEDTools intersect; (3) for narrowPeak files, selection of the top 10% regions based on signal value to focus on high-confidence regions; (4) sorting of resulting BED files by genomic coordinates; and (5) extraction of corresponding DNA sequences into FASTA format using the selected reference genome (options include mm10, GRCh37/38, hg19/38).

Motif enrichment analysis is performed using the MEME Suite’s AME tool with two background model options: either a shuffled background (preserving nucleotide frequencies of the input sequences) or a GC-content matched background generated by HOMER (v4.11). For the latter, peak regions are resized to 200 bp and HOMER’s background generation algorithm creates a set of matched control sequences. Results are processed to map motif IDs to their corresponding transcription factors using annotation files specific to each motif database, and the output is presented as a ranked table of enriched motifs with associated adjusted *p*-values, available for download in TSV format.

### User authentication

The Shiny application implements a comprehensive authentication and security framework to protect user data and manage API access. The system utilizes a multi-layered security approach with encrypted credential storage in an SQLite database. User passwords are hashed using the bcrypt algorithm with appropriate work factors, preventing storage of plaintext credentials. The authentication workflow incorporates several security mechanisms: account lockout after multiple failed login attempts with exponential backoff, mandatory password complexity requirements, and validation of unique usernames.

For secure management of OpenAI API keys, the application employs AES-CBC encryption with a distinct encryption key stored separately from the database. When a user uploads their API key, the system first validates the key’s authenticity by making a test request to the OpenAI API. Valid keys are either stored temporarily in the session or, at the user’s option, encrypted and persisted in the database with a randomly generated initialization vector (IV) prepended to the ciphertext and the entire payload encoded using Base64. This approach ensures API keys remain protected both in transit and at rest.

The application implements robust session security measures including Cross-Site Request Forgery (CSRF) token validation on all state-changing operations and an automatic session timeout that triggers after 300 seconds of inactivity.

### Setup of the web platform

The Shiny web-application was developed under R version 4.4.3 using the R base package shiny (version 1.7.4.1). To customize the user interface additional R packages, including shinythemes (version 1.2.0), shinyalerts (3.0.0), shinyWidgets (version 0.7.6), shinyfeedback (0.4.0), shinyglide (version 0.1.4), shinyjs (version 2.1.0), waiter (version 0.2.5) and shinyBS (version 0.61.1) were used. The app was containerized using docker (version 24.0.7) and was hosted on a Linux operating systems (Ubuntu 22.04.3 LTS).

## Supplementary information

Supplementary Information

## Data Availability

All data used and processed for DiReG is available at https://zenodo.org/api/records/15458798/files-archive. All code for the Shiny application and data preprocessing pipelines described in this manuscript is available in the GitHub repository (https://github.com/daisybio/DiReG).

## Code availability

All code for the Shiny application and data preprocessing pipelines described in this manuscript is available in the GitHub repository (https://github.com/daisybio/DiReG).
