# Supplementary material for: Navigating the landscape of direct cellular reprogramming with DiReG
Source: NPJ Syst Biol Appl. 2026 Feb 6;12:35. doi: 10.1038/s41540-026-00652-z (PMC12988218; doi:10.1038/s41540-026-00652-z)
Supplement: Supplementary file 1 — Supplementary Information [file 41540_2026_652_MOESM1_ESM.pdf]

Supplementary Figure 1: Expression profiles of HNF4A and FOXA family factors in donor and target tissues. Violin plots displaying the expression levels of HNF4A, FOXA1, FOXA2, and FOXA3 in human skin (representative of fibroblast donor tissue, left panel) compared to liver (representative of hepatocyte target tissue, right panel). Data is derived from Tabula Sapiens, showing log-transformed expression values. The plots demonstrate that these factors are highly expressed in the hepatic lineage while being virtually absent or highly reduced in the skin lineage, supporting their selection for transdifferentiation protocols.

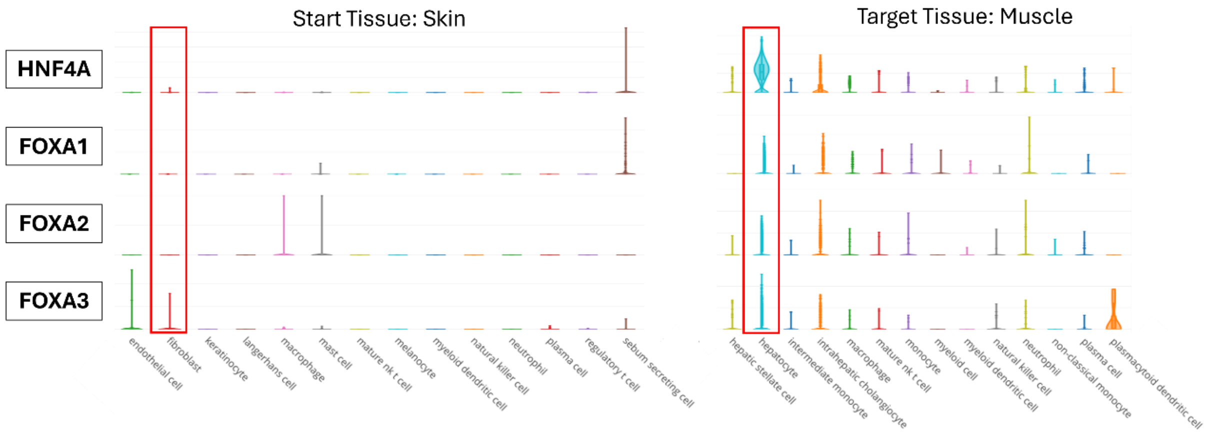

Supplementary Table S1: Curated database of direct reprogramming literature. A complete list of the 360 primary research publications included in the DiReG literature database. The table includes the PMCID, article title, list of authors, journal name, publication year, and DOI for each entry. These articles were identified via the NCBI E-utilities API using the query criteria described in the Methods section and filtered for relevance to cell biology using a Large Language Model (GPT-4o).

Pubmed_ID	Article_Title
40027671	Investigation of mitochondrial phenotypes in motor neurons derived by direct conversion of fibroblasts from familial ALS subjects.
39796127	Generation of Bona Fide Human Induced Trophoblast Stem Cells by Direct Reprogramming of Term Umbilical Cord Cells.
39617857	Correction: Expression levels and stoichiometry of Hnf1Î², Emx2, Pax8 and Hnf4Î± influence direct reprogramming of induced renal tubular epithelial cells.
39611044	Metabolic reprogramming via mitochondrial delivery for enhanced maturation of chemically induced cardiomyocyte-like cells.
39578834	Reprogrammed human lateral ganglionic eminence precursors generate striatal neurons and restore motor function in a rat model of Huntington's disease.
39565097	Non-apoptotic caspase events and Atf3 expression underlie direct neuronal differentiation of adult neural stem cells.

39540462	Age-associated metabolic and epigenetic barriers during direct reprogramming of mouse fibroblasts into induced cardiomyocytes.
39486406	Neural crest precursors from the skin are the primary source of directly reprogrammed neurons.
39347883	Expression levels and stoichiometry of Hnf1Î^{2}, Emx2, Pax8 and Hnf4 influence direct reprogramming of induced renal tubular epithelial cells.
39289870	EnhancerNet: a predictive model of cell identity dynamics through enhancer selection.
39279468	Epigenetic Dynamics in Reprogramming to Dopaminergic Neurons for Parkinson's Disease.
39272980	Comparing Viral Vectors and Fate Mapping Approaches for Astrocyte-to-Neuron Reprogramming in the Injured Mouse Cerebral Cortex.
39236715	3D model for human glia conversion into subtype-specific neurons, including dopamine neurons.
39227632	Suppression of PTBP1 in hippocampal astrocytes promotes neurogenesis and ameliorates recognition memory in mice with cerebral ischemia.
39185458	Modeling APOE Îµ4 familial Alzheimer's disease in directly converted 3D brain organoids.
39068473	Decoding single-cell molecular mechanisms in astrocyte-to-iN reprogramming via Ngn2- and Pax6-mediated direct lineage switching.
39014391	In vivo neural regeneration via AAV-NeuroD1 gene delivery to astrocytes in neonatal hypoxic-ischemic brain injury.
38961086	Generation of functional neurons from adult human mucosal olfactory ensheathing glia by direct lineage conversion.
38891029	Reprogramming Glioblastoma Cells into Non-Cancerous Neuronal Cells as a Novel Anti-Cancer Strategy.
38755186	Direct conversion of cardiac fibroblasts into endothelial-like cells using Sox17 and Erg.
38693086	Regulation of cardiac fibroblasts reprogramming into cardiomyocyte-like cells with a cocktail of small molecule compounds.
38530808	Macrophages suppress cardiac reprogramming of fibroblasts in vivo via IFN-mediated intercellular self-stimulating circuit.
38520033	Reprograming skin fibroblasts into Sertoli cells: a patient-specific tool to understand effects of genetic variants on gonadal development.
38513608	Arabidopsis ribosomal RNA processing meerling mutants exhibit suspensor-derived polyembryony due to direct reprogramming of the suspensor.
38374941	Corrigendum: Regeneration of the cerebral cortex by direct chemical reprogramming of macrophages into neuronal cells in acute ischemic stroke.
38273708	DIRECTEUR: transcriptome-based prediction of small molecules that replace transcription factors for direct cell conversion.
38229180	Vitamin C facilitates direct cardiac reprogramming by inhibiting reactive oxygen species.

38190040	Direct Reprogramming of Somatic Skin Cells from a Patient with Huntington's Disease into Striatal Neurons to Create Models of Pathology.
38132138	Exploring the Functional Heterogeneity of Directly Reprogrammed Neural Stem Cell-Derived Neurons via Single-Cell RNA Sequencing.
38102164	Transcription factor-mediated direct cellular reprogramming yields cell-type specific DNA methylation signature.
38077004	Proliferation history and transcription factor levels drive direct conversion.
37996395	Highly efficient conversion of mouse fibroblasts into functional hepatic cells under chemical induction.
37840130	Cardiomyocyte precursors generated by direct reprogramming and molecular beacon selection attenuate ventricular remodeling after experimental myocardial infarction.
37810261	Directly reprogrammed fragile X syndrome dorsal forebrain precursor cells generate cortical neurons exhibiting impaired neuronal maturation.
37771785	Detection of lineage-reprogramming efficiency of tumor cells in a 3D-printed liver-on-a-chip model.
37762156	Transplantation of Chemical Compound-Induced Cells from Human Fibroblasts Improves Locomotor Recovery in a Spinal Cord Injury Rat Model.
37749269	Single-cell lineage capture across genomic modalities with CellTag-multi reveals fate-specific gene regulatory changes.
37636590	Regeneration of the cerebral cortex by direct chemical reprogramming of macrophages into neuronal cells in acute ischemic stroke.
37553383	Transgene-free direct conversion of murine fibroblasts into functional muscle stem cells.
37540379	The efficient induction of human retinal ganglion-like cells provides a platform for studying optic neuropathies.
37377887	Reprogramming of Tumor-reactive Tumor-infiltrating Lymphocytes to Human-induced Pluripotent Stem Cells.
37357983	Reduction of Intracellular Tension and Cell Adhesion Promotes Open Chromatin Structure and Enhances Cell Reprogramming.
37072803	A robust reprogramming strategy for generating hepatocyte-like cells usable in pharmaco-toxicological studies.
37047770	Protocol Optimization for Direct Reprogramming of Primary Human Fibroblast into Induced Striatal Neurons.
36964318	Direct reprogramming of human fibroblasts into insulin-producing cells using transcription factors.
36963393	A miR-124-mediated post-transcriptional mechanism controlling the cell fate switch of astrocytes to induced neurons.
36910262	Small molecules fail to induce direct reprogramming of adult rat olfactory ensheathing glia to mature neurons.
36823979	Strategic Application of Epigenetic Regulators for Efficient Neuronal Reprogramming of Human Fibroblasts.

36819108	Rapid direct conversion of bovine non-adipogenic fibroblasts into adipocyte-like cells by a small-molecule cocktail.
36642416	Efficient Generation of Dopaminergic Neurons from Mouse Ventral Midbrain Astrocytes.
36611807	Impact of Mitochondrial A3243G Heteroplasmy on Mitochondrial Bioenergetics and Dynamics of Directly Reprogrammed MELAS Neurons.
36584685	Gene regulatory network reconfiguration in direct lineage reprogramming.
36575652	Erratum to: Acceleration of Mesenchymal-to-Epithelial Transition (MET) during Direct Reprogramming Using Natural Compounds.
36522156	HNF1B Alters an Evolutionarily Conserved Nephrogenic Program of Target Genes.
36499508	Direct Cardiac Epigenetic Reprogramming through Codelivery of 5'Azacytidine and miR-133a Nanoformulation.
36388958	Direct reprogramming of cardiomyocytes into cardiac Purkinje-like cells.
36289842	Direct Reprogramming of Mouse Subchondral Bone Osteoblasts into Chondrocyte-like Cells.
36284363	APOE Îµ4-dependent effects on the early amyloid pathology in induced neuronsÂ of patients with Alzheimer's disease.
36258029	Druggable transcriptomic pathways revealed in Parkinson's patient-derived midbrain neurons.
36253348	Retraction Note: Senescence impairs direct conversion of human somatic cells to neurons.
36231108	Global Transcriptional and Epigenetic Reconfiguration during Chemical Reprogramming of Human Retinal Pigment Epithelial Cells into Photoreceptor-like Cells.
36224763	Acceleration of Mesenchymal-to-Epithelial Transition (MET) during Direct Reprogramming Using Natural Compounds.
36142199	Genetic Mechanism Study of Auditory Phoenix Spheres and Transcription Factors Prediction for Direct Reprogramming by Bioinformatics.
36130499	A natural transdifferentiation event involving mitosis is empowered by integrating signaling inputs with conserved plasticity factors.
36106915	Probing cell identity hierarchies by fate titration and collision during direct reprogramming.
36102189	TBX20 Improves Contractility and Mitochondrial Function During Direct Human Cardiac Reprogramming.
36096985	Direct conversion of human umbilical cord mesenchymal stem cells into retinal pigment epithelial cells for treatment of retinal degeneration.
35887608	Modeling a Novel Variant of Glycogenosis IXa Using a Clonal Inducible Reprogramming System to Generate "Diseased" Hepatocytes for Accurate Diagnosis.
35883684	Induced Endothelial Cell-Integrated Liver Assembloids Promote Hepatic Maturation and Therapeutic Effect on Cholestatic Liver Fibrosis.
35877103	Chemical Replacement of Noggin with Dorsomorphin Homolog 1 for Cost-Effective Direct Neuronal Conversion.

35836274	Cellular direct conversion by cell penetrable OCT4-30Kc19 protein and BMP4 growth factor.
35832093	NOTCH1 signaling regulates the latent neurogenic program in adult reactive astrocytes after spinal cord injury.
35811849	Direct reprogramming of adult adipose-derived regenerative cells toward cardiomyocytes using six transcriptional factors.
35750047	Transcription factor-based direct conversion of human fibroblasts to functional astrocytes.
35705053	Genetic loss of function of Ptbp1 does not induce glia-to-neuron conversion in retina.
35688153	Fibroblast fate determination during cardiac reprogramming by remodeling of actin filaments.
35521891	Direct conversion from long-acting testosterone replacement therapy to Natesto allows for spermatogenesis resumption: Proof of concept.
35457029	Direct Reprograming of Mouse Fibroblasts into Dermal Papilla Cells via Small Molecules.
35409417	Direct Reprogramming and Induction of Human Dermal Fibroblasts to Differentiate into iPS-Derived Nucleus Pulposus-like Cells in 3D Culture.
35399519	Chemical Pretreatment Activated a Plastic State Amenable to Direct Lineage Reprogramming.
35385316	Integrative molecular roadmap for direct conversion of fibroblasts into myocytes and myogenic progenitor cells.
35373464	Parkinson's disease motor symptoms rescue by CRISPRa-reprogramming astrocytes into GABAergic neurons.
35351192	Small molecules facilitate single factor-mediated sweat gland cell reprogramming.
35269422	Cardiac Tissue-like 3D Microenvironment Enhances Route towards Human Fibroblast Direct Reprogramming into Induced Cardiomyocytes by microRNAs.
35220676	Direct Conversion of Human Endothelial Cells Into Liver Cancer-Forming Cells Using Nonintegrative Episomal Vectors.
35216271	Detection of Pathological Markers of Neurodegenerative Diseases following Microfluidic Direct Conversion of Patient Fibroblasts into Neurons.
35213232	Ultraefficient extracellular vesicle-guided direct reprogramming of fibroblasts into functional cardiomyocytes.
35127713	Generation of NKX2.5GFP Reporter Human iPSCs and Differentiation Into Functional Cardiac Fibroblasts.
35098228	Dynamics and Pathways of Chromosome Structural Organizations during Cell Transdifferentiation.
35027563	OCT4-induced oligodendrocyte progenitor cells promote remyelination and ameliorate disease.
34948246	Generation of Induced Nephron Progenitor-like Cells from Human Urine-Derived Cells.
34943958	Reprogramming Human Adult Fibroblasts into GABAergic Interneurons.
34936701	Distinct subcellular autophagy impairments in induced neurons from patients with Huntington's disease.

34889103	Hippo Pathway Effector Tead1 Induces Cardiac Fibroblast to Cardiomyocyte Reprogramming.
34746870	Direct conversion of adult fibroblasts into motor neurons.
34653405	Direct reprogramming of human Sertoli cells into male germline stem cells with the self-renewal and differentiation potentials via overexpressing DAZL/DAZ2/BOULE genes.
34540813	Osteogenic Response to Polysaccharide Nanogel Sheets of Human Fibroblasts After Conversion Into Functional Osteoblasts by Direct Phenotypic Cell Reprogramming.
34502264	Cell-Permeable Oct4 Gene Delivery Enhances Stem Cell-like Properties of Mouse Embryonic Fibroblasts.
34490268	Restoration of Visual Function and Cortical Connectivity After Ischemic Injury Through NeuroD1-Mediated Gene Therapy.
34332145	Direct reprogramming induces vascular regeneration post muscle ischemic injury.
34289357	Heterogeneity of neurons reprogrammed from spinal cord astrocytes by the proneural factors Ascl1 and Neurogenin2.
34222247	Stepwise Induction of Inner Ear Hair Cells From Mouse Embryonic Fibroblasts via Mesenchymal- to-Epithelial Transition and Formation of Otic Epithelial Cells.
34215318	High-efficiency c-Myc-mediated induction of functional hepatoblasts from the human umbilical cord mesenchymal stem cells.
34208436	Transdifferentiation of Human Fibroblasts into Skeletal Muscle Cells: Optimization and Assembly into Engineered Tissue Constructs through Biological Ligands.
34151292	A protocol for transdifferentiation of human cardiac fibroblasts into endothelial cells via activation of innate immunity.
34136075	Disease modifying treatment of spinal cord injury with directly reprogrammed neural precursor cells in non-human primates.
34113099	Highly Efficient MicroRNA Delivery Using Functionalized Carbon Dots for Enhanced Conversion of Fibroblasts to Cardiomyocytes.
34088673	Conversion of mouse embryonic fibroblasts into neural crest cells and functional corneal endothelia by defined small molecules.
34087994	Inhibition of EZH2 primes the cardiac gene activation via removal of epigenetic repression during human direct cardiac reprogramming.
33927320	Direct conversion of porcine primary fibroblasts into hepatocyte-like cells.
33910058	Age-dependent instability of mature neuronal fate in induced neurons from Alzheimer's patients.
33854891	Asymmetric Cell Division of Fibroblasts is An Early Deterministic Step to Generate Elite Cells during Cell Reprogramming.
33841125	Conversion of Reactive Astrocytes to Induced Neurons Enhances Neuronal Repair and Functional Recovery After Ischemic Stroke.
33803331	Direct Conversion of Human Fibroblasts into Adipocytes Using a Novel Small Molecular Compound: Implications for Regenerative Therapy for Adipose Tissue Defects.

33770499	One-step Reprogramming of Human Fibroblasts into Oligodendrocyte-like Cells by SOX10, OLIG2, and NKX6.2.
33718351	Sall4 and Myocd Empower Direct Cardiac Reprogramming From Adult Cardiac Fibroblasts After Injury.
33665626	Protocol for generating human induced neural progenitor cells from immobilized adult peripheral blood.
33642376	In vivo direct reprogramming as a therapeutic strategy for brain and retina repair.
33605072	Cyclic stretching boosts microRNA-499 to regulate Bcl-2 via microRNA-208a in atrial fibroblasts.
33574458	Direct reprogramming of oligodendrocyte precursor cells into GABAergic inhibitory neurons by a single homeodomain transcription factor Dlx2.
33473142	Direct reprogramming of epithelial cell rests of malassez into mesenchymal-like cells by epigenetic agents.
33452356	A quantitative model of cellular decision making in direct neuronal reprogramming.
33400690	Direct conversion of osteosarcoma to adipocytes by targeting TNIK.
33375083	Direct Neuronal Reprogramming of Common Marmoset Fibroblasts by ASCL1, microRNA-9/9*, and microRNA-124 Overexpression.
33314575	Directly converted astrocytes retain the ageing features of the donor fibroblasts and elucidate the astrocytic contribution to human CNS health and disease.
33293623	Dermal fibroblast-like cells reprogrammed directly from adipocytes in mouse.
33250718	Development of Neuroregenerative Gene Therapy to Reverse Glial Scar Tissue Back to Neuron-Enriched Tissue.
33202244	CRISPR-Mediated Induction of Neuron-Enriched Mitochondrial Proteins Boosts Direct Glia-to-Neuron Conversion.
33182669	Direct Conversion of Human Stem Cell-Derived Glial Progenitor Cells into GABAergic Interneurons.
33129359	Direct conversion of human fibroblasts into dopaminergic neuron-like cells using small molecules and protein factors.
33096050	Quick Commitment and Efficient Reprogramming Route of Direct Induction of Retinal Ganglion Cell-like Neurons.
33093268	Direct reprogramming of fibroblasts into diverse lineage cells by DNA demethylation followed by differentiating cultures.
33087715	Direct reprogramming of human umbilical vein- and peripheral blood-derived endothelial cells into hepatic progenitor cells.
33064855	Generation of metabolically functional hepatocyte-like cells from dedifferentiated fat cells by Foxa2, Hnf4a and Sall1 transduction.
33037790	Forskolin rapidly enhances neuron-like morphological change of directly induced-neuronal cells from neurofibromatosis type 1 patients.
32976765	Direct Reprogramming of Human Fetal- and Stem Cell-Derived Glial Progenitor Cells into Midbrain Dopaminergic Neurons.
32953733	The combination of forskolin and VPA increases gene expression efficiency to the hypoxia/neuron-specific system.

32896271	Direct reprogramming of human smooth muscle and vascular endothelial cells reveals defects associated with aging and Hutchinson-Gilford progeria syndrome.
32784741	Neuroprotective Effects of Cryptotanshinone in a Direct Reprogramming Model of Parkinson's Disease.
32602462	Generation of inner ear hair cells by direct lineage conversion of primary somatic cells.
32586386	Directly induced human Schwann cell precursors as a valuable source of Schwann cells.
32582662	MicroRNA-Mediated Direct Reprogramming of Human Adult Fibroblasts Toward Cardiac Phenotype.
32571478	Sequentially induced motor neurons from human fibroblasts facilitate locomotor recovery in a rodent spinal cord injury model.
32500803	Enhanced Generation of Induced Cardiomyocytes Using a Small-Molecule Cocktail to Overcome Barriers to Cardiac Cellular Reprogramming.
32341358	The H2B ubiquitin-protein ligase RNF40 is required for somatic cell reprogramming.
32276654	Phenotype instability of hepatocyte-like cells produced by direct reprogramming of mesenchymal stromal cells.
32274291	Direct Conversion of Human Dermal Fibroblasts into Cardiomyocyte-Like Cells Using CiCMC Nanogels Coupled with Cardiac Transcription Factors and a Nucleoside Drug.
32201555	Ectopic transient overexpression of OCT-4 facilitates BMP4-induced osteogenic transdifferentiation of human umbilical vein endothelial cells.
32123700	Conversion of mesenchymal stem cells into a canine hepatocyte-like cells by Foxa1 and Hnf4a.
32114739	Robust and Reproducible Generation of Induced Neural Stem Cells from Human Somatic Cells by Defined Factors.
32111895	A developed serum-free medium and an optimized chemical cocktail for direct conversion of human dermal fibroblasts into brown adipocytes.
32084387	High-Resolution Dissection of Chemical Reprogramming from Mouse Embryonic Fibroblasts into Fibrocartilaginous Cells.
32071715	Reprogrammed astrocytes display higher neurogenic competence, migration ability and cell death resistance than reprogrammed fibroblasts.
31979018	Isoform Specific Effects of Mef2C during Direct Cardiac Reprogramming.
31940860	Cytokine Directed Chondroblast Trans-Differentiation: JAK Inhibition Facilitates Direct Reprogramming of Fibroblasts to Chondroblasts.
31849638	Very Low Efficiency of Direct Reprogramming of Astrocytes Into Neurons in the Brains of Young and Aged Mice After Cerebral Ischemia.
31784643	Glutamine/glutamate metabolism rewiring in reprogrammed human hepatocyte-like cells.
31723223	Direct Conversion of Human Urine Cells to Neurons by Small Molecules.

31712992	Direct conversion of human fibroblasts into therapeutically active vascular wall-typical mesenchymal stem cells.
31685531	Developmental and cellular age direct conversion of CD4+ T cells into RORÎ³+ or Helios+ colon Treg cells.
31685034	Direct conversion of human fibroblast to hepatocytes using a single inducible polycistronic vector.
31660065	Cardiac-mimetic cell-culture system for direct cardiac reprogramming.
31637157	Exosome-Guided Phenotypic Switch of M1 to M2 Macrophages for Cutaneous Wound Healing.
31631018	Rapid and Efficient Conversion of Human Fibroblasts into Functional Neurons by Small Molecules.
31591186	Yorkie and JNK revert syncytial muscles into myoblasts during Org-1-dependent lineage reprogramming.
31554870	Direct conversion of fibroblasts into urothelial cells that may be recruited to regenerating mucosa of injured urinary bladder.
31551693	Neurons Induced From Fibroblasts of c9ALS/FTD Patients Reproduce the Pathology Seen in the Central Nervous System.
31501413	Direct neuronal reprogramming of olfactory ensheathing cells for CNS repair.
31489945	mRNA-Driven Generation of Transgene-Free Neural Stem Cells from Human Urine-Derived Cells.
31474031	Direct Reprogramming to Human Induced Neuronal Progenitors from Fibroblasts of Familial and Sporadic Parkinson's Disease Patients.
31419975	Exosomes released from neural progenitor cells and induced neural progenitor cells regulate neurogenesis through miR-21a.
31404112	Oct4 and Hnf4Î±-induced hepatic stem cells ameliorate chronic liver injury in liver fibrosis model.
31358888	In vivo direct reprogramming of glial linage to mature neurons after cerebral ischemia.
31347791	Chemotherapy-Induced Neuropathy and Drug Discovery Platform Using Human Sensory Neurons Converted Directly from Adult Peripheral Blood.
31324753	Efficient exogenous DNA-free reprogramming with suicide gene vectors.
31248447	Generation of functional dopaminergic neurons from human spermatogonial stem cells to rescue parkinsonian phenotypes.
31236114	Pharmacological Transdifferentiation of Human Nasal Olfactory Stem Cells into Dopaminergic Neurons.
31212628	Inhibition of Glioma Development by ASCL1-Mediated Direct Neuronal Reprogramming.
31196173	Examining the fundamental biology of a novel population of directly reprogrammed human neural precursor cells.
31130844	Corrigendum: Potentials of Cellular Reprogramming as a Novel Strategy for Neuroregeneration.
31099332	Chemical modulation of transcriptionally enriched signaling pathways to optimize the conversion of fibroblasts into neurons.
31089332	Metastable Reprogramming State of Single Transcription Factor-Derived Induced Hepatocyte-Like Cells.

31023000	Hybrid Nanofiber Scaffold-Based Direct Conversion of Neural Precursor Cells/Dopamine Neurons.
30962586	Comparison of three congruent patient-specific cell types for the modelling of a human genetic Schwann-cell disorder.
30894517	Direct reprogramming of epidermal cells toward sweat gland-like cells by defined factors.
30731320	Generation of Genetically Stable Human Direct-Conversion-Derived Neural Stem Cells Using Quantity Control of Proto-oncogene Expression.
30713039	Region-Restrict Astrocytes Exhibit Heterogeneous Susceptibility to Neuronal Reprogramming.
30675392	Direct conversion of pig fibroblasts to chondrocyte-like cells by c-Myc.
30644360	Global DNA methylation remodeling during direct reprogramming of fibroblasts to neurons.
30635054	The therapeutic potential of induced hepatocyte-like cells generated by direct reprogramming on hepatic fibrosis.
30539819	Plasmid-based generation of neural cells from human fibroblasts using non-integrating episomal vectors.
30518857	Single-cell mapping of lineage and identity in direct reprogramming.
30512203	Endothelial Differentiation G Protein-Coupled Receptor 5 Plays an Important Role in Induction and Maintenance of Pluripotency.
30450440	Conversion of adult human fibroblasts into neural precursor cells using chemically modified mRNA.
30410751	Direct conversion of mouse astrocytes into neural progenitor cells and specific lineages of neurons.
30361649	Nanogel tectonic porous 3D scaffold for direct reprogramming fibroblasts into osteoblasts and bone regeneration.
30349553	5-Aza Exposure Improves Reprogramming Process Through Embryoid Body Formation in Human Gingival Stem Cells.
30327781	Nonintegrating Direct Conversion Using mRNA into Hepatocyte-Like Cells.
30318292	Phenotypic Reprogramming of Striatal Neurons into Dopaminergic Neuron-like Cells in the Adult Mouse Brain.
30063705	High-resolution transcriptional dissection of in vivo Atoh1-mediated hair cell conversion in mature cochleae identifies Isl1 as a co-reprogramming factor.
30030434	Direct reprogramming of fibroblasts into neural stem cells by single non-neural progenitor transcription factor Ptf1a.
30018471	Generation of dopamine neuronal-like cells from induced neural precursors derived from adult human cells by non-viral expression of lineage factors.
29979748	Transcription factor induced conversion of human fibroblasts towards the hair cell lineage.
29959867	Hepatocyte-like cells generated by direct reprogramming from murine somatic cells can repopulate decellularized livers.
29937717	Direct Reprogramming of Adult Human Somatic Stem Cells Into Functional Neurons Using Sox2, Ascl1, and Neurog2.

29915193	Direct pericyte-to-neuron reprogramming via unfolding of a neural stem cell-like program.
29910772	Rosiglitazone and a Î²3-Adrenoceptor Agonist Are Both Required for Functional Browning of White Adipocytes in Culture.
29909688	Single-Factor SOX2 Mediates Direct Neural Reprogramming of Human Mesenchymal Stem Cells via Transfection of In Vitro Transcribed mRNA.
29880807	Publisher Correction: Direct reprogramming of fibroblasts into skeletal muscle progenitor cells by transcription factors enriched in undifferentiated subpopulation of satellite cells.
29855543	Direct phenotypic conversion of human fibroblasts into functional osteoblasts triggered by a blockade of the transforming growth factor-Î² signal.
29743891	A Loss of Function Screen of Epigenetic Modifiers and Splicing Factors during Early Stage of Cardiac Reprogramming.
29742392	Direct Reprogramming of Mouse Fibroblasts into Functional Skeletal Muscle Progenitors.
29734659	S-phase Synchronization Facilitates the Early Progression of Induced-Cardiomyocyte Reprogramming through Enhanced Cell-Cycle Exit.
29695818	Author Correction: Direct reprogramming of fibroblasts into skeletal muscle progenitor cells by transcription factors enriched in undifferentiated subpopulation of satellite cells.
29606616	Direct Conversion of Mouse Fibroblasts into Cholangiocyte Progenitor Cells.
29507336	Direct conversion of injury-site myeloid cells to fibroblast-like cells of granulation tissue.
29497074	Metabolic characterization of directly reprogrammed renal tubular epithelial cells (iRECs).
29492404	Direct Reprogramming of Spiral Ganglion Non-neuronal Cells into Neurons: Toward Ameliorating Sensorineural Hearing Loss by Gene Therapy.
29474672	Leptomeninges-Derived Induced Pluripotent Stem Cells and Directly Converted Neurons From Autopsy Cases With Varying Neuropathologic Backgrounds.
29352247	High content analysis identifies unique morphological features of reprogrammed cardiomyocytes.
29317869	Directly Converted Human Fibroblasts Mature to Neurons and Show Long-Term Survival in Adult Rodent Hippocampus.
29311646	Generation of Functional Dopaminergic Neurons from Reprogramming Fibroblasts by Nonviral-based Mesoporous Silica Nanoparticles.
29260008	Functional validation and expression analysis of myotubes converted from skin fibroblasts using a simple direct reprogramming strategy.
29207072	Transcription factor TBX18 promotes adult rat bone mesenchymal stem cell differentiation to biological pacemaker cells.
29192290	Direct conversion of human fibroblasts into hepatocyte-like cells by ATF5, PROX1, FOXA2, FOXA3, and HNF4A transduction.
29185460	Reprogramming to pluripotency does not require transition through a primitive streak-like state.

29174331	Constitutively Active SMAD2/3 Are Broad-Scope Potentiators of Transcription-Factor-Mediated Cellular Reprogramming.
29163034	Direct Reprogramming Rather than iPSC-Based Reprogramming Maintains Aging Hallmarks in Human Motor Neurons.
29156730	Generation of patient specific human neural stem cells from Niemann-Pick disease type C patient-derived fibroblasts.
29137640	Direct reprogramming of mouse fibroblasts into neural cells via Porphyra yezoensis polysaccharide based high efficient gene co-delivery.
29066822	Dysregulated gene expressions of MEX3D, FOS and BCL2 in human induced-neuronal (iN) cells from NF1 patients: a pilot study.
28982760	ZNF281 enhances cardiac reprogramming by modulating cardiac and inflammatory gene expression.
28982679	Evaluation of human dermal fibroblasts directly reprogrammed to adipocyte-like cells as a metabolic disease model.
28973471	Mechanisms of transcription factor-mediated direct reprogramming of mouse embryonic stem cells to trophoblast stem-like cells.
28962665	Direct conversion of human fibroblasts to functional excitatory cortical neurons integrating into human neural networks.
28874788	Comprehensive transcriptome mining of the direct conversion of mesodermal cells.
28844658	Direct Reprogramming of Resident NG2 Glia into Neurons with Properties of Fast-Spiking Parvalbumin-Containing Interneurons.
28844127	Direct Conversion of Human Umbilical Cord Blood into Induced Neural Stem Cells with SOX2 and HMGA2.
28825623	Single-Construct Polycistronic Doxycycline-Inducible Vectors Improve Direct Cardiac Reprogramming and Can Be Used to Identify the Critical Timing of Transgene Expression.
28808339	Direct reprogramming of fibroblasts into skeletal muscle progenitor cells by transcription factors enriched in undifferentiated subpopulation of satellite cells.
28796841	Single cell qPCR reveals that additional HAND2 and microRNA-1 facilitate the early reprogramming progress of seven-factor-induced human myocytes.
28655922	Direct conversion of human fibroblasts to brown adipocytes by small chemical compounds.
28646119	REST suppression mediates neural conversion of adult human fibroblasts via microRNA-dependent and -independent pathways.
28605832	Comparison of Reprogramming Methods for Generation of Induced-Oligodendrocyte Precursor Cells.
28587331	Directly reprogramming fibroblasts into adipogenic, neurogenic and hepatogenic differentiation lineages by defined factors.
28352551	An easy method for preparation of Cre-loxP regulated fluorescent adenoviral expression vectors and its application for direct reprogramming into hepatocytes.
28344997	Simple Derivation of Spinal Motor Neurons from ESCs/iPSCs Using Sendai Virus Vectors.

28344001	Inducible and Deterministic Forward Programming of Human Pluripotent Stem Cells into Neurons, Skeletal Myocytes, and Oligodendrocytes.
28327614	Partial Reprogramming of Pluripotent Stem Cell-Derived Cardiomyocytes into Neurons.
28321434	Transient CREB-mediated transcription is key in direct neuronal reprogramming.
28295042	Generation of non-viral, transgene-free hepatocyte like cells with piggyBac transposon.
28213969	Highly Efficient Neural Conversion of Human Pluripotent Stem Cells in Adherent and Animal-Free Conditions.
28186702	Direct Conversion of Human Fibroblasts into Schwann Cells that Facilitate Regeneration of Injured Peripheral Nerve In Vivo.
28169300	Two factor-based reprogramming of rodent and human fibroblasts into Schwann cells.
28110217	Induced dopaminergic neurons: A new promise for Parkinson's disease.
28099929	Modeling the phenotype of spinal muscular atrophy by the direct conversion of human fibroblasts to motor neurons.
28042541	Rational Development of A Polycistronic Plasmid with A CpG-Free Bacterial Backbone as A Potential Tool for Direct Reprogramming.
28017657	Direct Reprogramming of Mouse Fibroblasts toward Leydig-like Cells by Defined Factors.
27941896	Tissue-engineered 3-dimensional (3D) microenvironment enhances the direct reprogramming of fibroblasts into cardiomyocytes by microRNAs.
27940274	Direct Conversion of Human Fibroblasts into Neural Progenitors Using Transcription Factors Enriched in Human ESC-Derived Neural Progenitors.
27930352	MiR-590 Promotes Transdifferentiation of Porcine and Human Fibroblasts Toward a Cardiomyocyte-Like Fate by Directly Repressing Specificity Protein 1.
27902735	Role of Hepatic-Specific Transcription Factors and Polycomb Repressive Complex 2 during Induction of Fibroblasts to Hepatic Fate.
27857203	Rapid and efficient CRISPR/Cas9 gene inactivation in human neurons during human pluripotent stem cell differentiation and direct reprogramming.
27822179	Plasmid-Based Generation of Induced Neural Stem Cells from Adult Human Fibroblasts.
27651888	Direct reprogramming of urine-derived cells with inducible MyoD for modeling human muscle disease.
27630998	The Function of the MEF2 Family of Transcription Factors in Cardiac Development, Cardiogenomics, and Direct Reprogramming.
27606336	Adult neural stem cell behavior underlying constitutive and restorative neurogenesis in zebrafish.
27602403	Small molecule-driven direct conversion of human pluripotent stem cells into functional osteoblasts.
27569063	Expandable and Rapidly Differentiating Human Induced Neural Stem Cell Lines for Multiple Tissue Engineering Applications.

27488544	Significant improvement of direct reprogramming efficacy of fibroblasts into progenitor endothelial cells by ETV2 and hypoxia.
27473056	Direct conversion of mouse embryonic fibroblasts into functional keratinocytes through transient expression of pluripotency-related genes.
27460218	Silencing of hepatic fate-conversion factors induce tumorigenesis in reprogrammed hepatic progenitor-like cells.
27396343	Small-Molecule-Based Lineage Reprogramming Creates Functional Astrocytes.
27387763	Loss of tumorigenic potential upon transdifferentiation from keratinocytic into melanocytic lineage.
27281220	Dissecting direct reprogramming from fibroblast to neuron using single-cell RNA-seq.
27187823	Reprogramming of Pancreatic Exocrine Cells AR42J Into Insulin-producing Cells Using mRNAs for Pdx1, Ngn3, and MafA Transcription Factors.
27175211	Protocol: a method to study the direct reprogramming of lateral root primordia to fertile shoots.
27170256	In vitro transdifferentiation of human peripheral blood mononuclear cells to photoreceptor-like cells.
27148066	A "Hit and Run" Approach to Inducible Direct Reprogramming of Astrocytes to Neural Stem Cells.
27112843	Human somatic cells subjected to genetic induction with six germ line-related factors display meiotic germ cell-like features.
27110916	Sequential regulatory loops as key gatekeepers for neuronal reprogramming in human cells.
27110487	White-to-brite conversion in human adipocytes promotes metabolic reprogramming towards fatty acid anabolic and catabolic pathways.
27081470	Therapeutic transdifferentiation of human fibroblasts into endothelial cells using forced expression of lineage-specific transcription factors.
27077806	Cardiac mesenchymal progenitors differentiate into adipocytes via Klf4 and c-Myc.
27052315	Conversion of Human Fibroblasts to Stably Self-Renewing Neural Stem Cells with a Single Zinc-Finger Transcription Factor.
27019637	Direct Reprogramming of Human Amniotic Fluid Stem Cells by OCT4 and Application in Repairing of Cerebral Ischemia Damage.
26981072	Direct reprogramming of somatic cells into neural stem cells or neurons for neurological disorders.
26975336	Selenium Augments microRNA Directed Reprogramming of Fibroblasts to Cardiomyocytes via Nanog.
26873953	Gene array analysis of neural crest cells identifies transcription factors necessary for direct conversion of embryonic fibroblasts into neural crest cells.
26830441	Therapeutically engineered induced neural stem cells are tumour-homing and inhibit progression of glioblastoma.
26788068	Direct Reprogramming of Mouse Fibroblasts to Neural Stem Cells by Small Molecules.

26719791	Reprogramming somatic cells to cells with neuronal characteristics by defined medium both in vitro and in vivo.
26681949	Cardiac Niche Influences the Direct Reprogramming of Canine Fibroblasts into Cardiomyocyte-Like Cells.
26659182	The histone chaperone CAF-1 safeguards somatic cell identity.
26639555	Cell cycle and p53 gate the direct conversion of human fibroblasts to dopaminergic neurons.
26634071	FGF8 is Essential for Functionality of Induced Neural Precursor Cell-derived Dopaminergic Neurons.
26535892	SOX2 and SOX2-MYC Reprogramming Process of Fibroblasts to the Neural Stem Cells Compromised by Senescence.
26503743	Generation of integration-free induced hepatocyte-like cells from mouse fibroblasts.
26354680	High-efficiency reprogramming of fibroblasts into cardiomyocytes requires suppression of pro-fibrotic signalling.
26306155	Lower Oncogenic Potential of Human Mesenchymal Stem Cells Derived from Cord Blood Compared to Induced Pluripotent Stem Cells.
26302234	Enhanced efficiency of genetic programming toward cardiomyocyte creation through topographical cues.
26292833	Direct reprogramming of mouse fibroblasts into cardiomyocytes with chemical cocktails.
26224135	Selective Generation of Dopaminergic Precursors from Mouse Fibroblasts by Direct Lineage Conversion.
26216300	Direct conversion of human fibroblasts to induced serotonergic neurons.
26060345	Highly efficient direct conversion of human fibroblasts to neuronal cells by chemical compounds.
26030913	MicroRNA-Mediated In Vitro and In Vivo Direct Conversion of Astrocytes to Neuroblasts.
25949812	Direct reprogramming of induced neural progenitors: a new promising strategy for AD treatment.
25919922	Direct conversion of human myoblasts into brown-like adipocytes by engineered super-active PPARÎ³.
25890371	Predicting involvement of polycomb repressive complex 2 in direct conversion of mouse fibroblasts into induced neural stem cells.
25870845	Brief azacytidine step allows the conversion of suspension human fibroblasts into neural progenitor-like cells.
25834424	Peptide-enhanced mRNA transfection in cultured mouse cardiac fibroblasts and direct reprogramming towards cardiomyocyte-like cells.
25754206	Direct reprogramming of human bone marrow stromal cells into functional renal cells using cell-free extracts.
25556566	Direct conversion of fibroblasts into functional astrocytes by defined transcription factors.
25510211	Direct conversion of mouse and human fibroblasts to functional melanocytes by defined factors.
25494287	Enhanced MyoD-induced transdifferentiation to a myogenic lineage by fusion to a potent transactivation domain.

25466247	Direct reprogramming of murine fibroblasts to hematopoietic progenitor cells.
25333522	Non-genetic direct reprogramming and biomimetic platforms in a preliminary study for adipose-derived stem cells into corneal endothelia-like cells.
25309947	Embedding the Future of Regenerative Medicine into the Open Epigenomic Landscape of Pluripotent Human Embryonic Stem Cells.
25275533	Direct reprogramming of Huntington's disease patient fibroblasts into neuron-like cells leads to abnormal neurite outgrowth, increased cell death, and aggregate formation.
25254342	Generation of induced neuronal cells by the single reprogramming factor ASCL1.
25238487	PIWI proteins are dispensable for mouse somatic development and reprogramming of fibroblasts into pluripotent stem cells.
24963715	Direct reprogramming of human fibroblasts to hepatocyte-like cells by synthetic modified mRNAs.
24926434	Constraining the Pluripotent Fate of Human Embryonic Stem Cells for Tissue Engineering and Cell Therapy - The Turning Point of Cell-Based Regenerative Medicine.
24694048	Reprogramming non-human primate somatic cells into functional neuronal cells by defined factors.
24586958	Inhibition of TGFÎ^{2} signaling increases direct conversion of fibroblasts to induced cardiomyocytes.
24474194	Direct conversion of human fibroblasts into retinal pigment epithelium-like cells by defined factors.
29805851	Reversibility of cellular aging by reprogramming through an embryonic-like state: a new paradigm for human cell rejuvenation.
24319660	Direct reprogramming of human fibroblasts toward a cardiomyocyte-like state.
24232094	A set of microRNAs mediate direct conversion of human umbilical cord lining-derived mesenchymal stem cells into hepatocytes.
24098810	Efficient production of retroviruses using PLGA/bPEI-DNA nanoparticles and application for reprogramming somatic cells.
23974433	Environmental impact on direct neuronal reprogramming in vivo in the adult brain.
23584611	Transcription factor-mediated reprogramming of fibroblasts to expandable, myelinogenic oligodendrocyte progenitor cells.
23582880	A novel model of urinary tract differentiation, tissue regeneration, and disease: reprogramming human prostate and bladder cells into induced pluripotent stem cells.
23344148	Nonviral direct conversion of primary mouse embryonic fibroblasts to neuronal cells.
23336074	Wnt/Î^{2}-catenin signaling cell-autonomously converts non-hepatic endodermal cells to a liver fate.
23326632	RETRACTION: A therapeutic method for the direct reprogramming of human liver cancer cells with only chemicals.

23102146	HIRA dependent H3.3 deposition is required for transcriptional reprogramming following nuclear transfer to Xenopus oocytes.
22912920	Reversal of aberrant cancer methylome and transcriptome upon direct reprogramming of lung cancer cells.
22833560	Placenta to cartilage: direct conversion of human placenta to chondrocytes with transformation by defined factors.
22715383	Calcium dependent CAMTA1 in adult stem cell commitment to a myocardial lineage.
22708059	Molecular mechanisms of induced pluripotency.
22426197	Direct reprogramming of human astrocytes into neural stem cells and neurons.
22355790	A therapeutic method for the direct reprogramming of human liver cancer cells with only chemicals.
24693194	Non-Viral Generation of Neural Precursor-like Cells from Adult Human Fibroblasts.
22174877	Efficient conversion of astrocytes to functional midbrain dopaminergic neurons using a single polycistronic vector.
22105488	Direct reprogramming of human fibroblasts into dopaminergic neuron-like cells.
22046312	Efficient generation of fully reprogrammed human iPS cells via polycistronic retroviral vector and a new cocktail of chemical compounds.
22028025	Reprogramming capacity of Nanog is functionally conserved in vertebrates and resides in a unique homeodomain.
21977043	Hepatic differentiation of murine disease-specific induced pluripotent stem cells allows disease modelling in vitro.
21533226	Reprogramming of embryonic human fibroblasts into fetal hematopoietic progenitors by fusion with human fetal liver CD34+ cells.
20948840	Reconstructing blood from induced pluripotent stem cells.
20352099	Direct reprogramming of rat neural precursor cells and fibroblasts into pluripotent stem cells.
20107439	Direct conversion of fibroblasts to functional neurons by defined factors.
19898493	Direct cell reprogramming is a stochastic process amenable to acceleration.
19578358	miR-145 and miR-143 regulate smooth muscle cell fate and plasticity.
19252477	Virus-free induction of pluripotency and subsequent excision of reprogramming factors.
14581455	Nodal-dependent Cripto signaling promotes cardiomyogenesis and redirects the neural fate of embryonic stem cells.
